# Genome-scale CRISPR screens identify PTGES3 as a direct modulator of androgen receptor function in advanced prostate cancer

**DOI:** 10.1038/s41588-025-02388-8

**Published:** 2025-11-05

**Authors:** Haolong Li, James E. Melnyk, Becky Xu Hua Fu, Raunak Shrestha, Meng Zhang, Martin Sjöström, Siyu Feng, Jasmine A. Anderson, Wanting Han, Lisa N. Chesner, Hyun Jin Shin, Tatyanah Farsh, Humberto J. Suarez, Seema Nath, Jonathan Chou, Rajdeep Das, Emily A. Egusa, Marsha Calvert, Audrey Kishishita, Abhilash Barpanda, Jun Zhu, Ashutosh Maheshwari, William S. Chen, Mohammed Alshalalfa, Aidan Winters, Junjie T. Hua, Tianyi Liu, Elai Davicioni, Arun P. Wiita, Bradley A. Stohr, Javed Siddiqui, Bo Huang, Eric J. Small, Kevan M. Shokat, Peter S. Nelson, David A. Quigley, Elizabeth V. Wasmuth, Luke A. Gilbert, Felix Y. Feng

**Affiliations:** 1 Human Biology Division, Fred Hutchinson Cancer Center, Seattle, WA USA; 2 Helen Diller Family Comprehensive Cancer Center, University of California San Francisco, San Francisco, CA USA; 3 Department of Radiation Oncology, University of California San Francisco, San Francisco, CA USA; 4 Department of Cellular and Molecular Pharmacology, University of California San Francisco, San Francisco, CA USA; 5 Arc Institute, Palo Alto, CA USA; 6 Department of Urology, University of California San Francisco, San Francisco, CA USA; 7 Department of Urology, Emory University, Atlanta, GA USA; 8 Department of Clinical Sciences Lund, Division of Oncology and Pathology, Faculty of Medicine, Lund University, Lund, Sweden; 9 The UC Berkeley-UCSF Graduate Program in Bioengineering, San Francisco, CA USA; 10 Department of Biochemistry and Structural Biology, Greehey Children’s Cancer Research Institute, University of Texas Health Science Center at San Antonio, San Antonio, TX USA; 11 Department of Medicine, Division of Hematology and Oncology, University of California San Francisco, San Francisco, CA USA; 12 Department of Laboratory Medicine, University of California San Francisco, San Francisco, CA USA; 13 Graduate Program in Biological and Medical Informatics, University of California San Francisco, San Francisco, CA USA; 14 Veracyte Inc, San Diego, CA USA; 15 Department of Pathology, University of California San Francisco, San Francisco, CA USA; 16 Department of Pathology, University of Michigan, Ann Arbor, MI USA; 17 Department of Pharmaceutical Chemistry, University of CaliforniaSan Francisco, San Francisco, CA USA; 18 Department of Biochemistry and Biophysics, University of California San Francisco, San Francisco, CA USA; 19 Chan Zuckerberg Biohub San Francisco, San Francisco, CA USA; 20 Howard Hughes Medical Institute, University of California San Francisco, San Francisco, CA USA; 21 Department of Epidemiology and Biostatistics, University of California San Francisco, San Francisco, CA USA

**Keywords:** Prostate cancer, Cancer

## Abstract

The androgen receptor (AR) is a critical driver of prostate cancer (PCa). Here, to study regulators of AR protein levels and oncogenic activity, we developed a live-cell quantitative endogenous AR fluorescent reporter. Leveraging this AR reporter, we performed genome-scale CRISPRi flow cytometry sorting screens to systematically identify genes that modulate AR protein levels. We identified and validated known AR protein regulators, including HOXB13 and GATA2, and also unexpected top hits including PTGES3—a poorly characterized gene in PCa. PTGES3 repression resulted in loss of AR protein, cell-cycle arrest and cell death in AR-driven PCa models. Clinically, analysis of PCa data demonstrates that PTGES3 expression is associated with AR-directed therapy resistance. Mechanistically, we show PTGES3 binds directly to AR, regulates AR protein stability and is necessary for AR function in the nucleus at AR target genes. PTGES3 represents a potential therapeutic target for overcoming known mechanisms of resistance to existing AR-directed therapies in PCa.

## Abstract (web summary)

Genome-wide CRISPRi screens for modulators of androgen receptor (AR) protein levels using live-cell quantitative endogenous AR fluorescence reporters identify PTGES3 as a new regulator of AR stability and function in prostate cancer.

## Main

The androgen receptor (AR) is a central driver of tumorigenesis and disease progression in prostate cancer (PCa)^1,2^. Most PCas express AR throughout the course of the disease^2–5^, and AR has been shown to promote transcriptional programs governing critical oncogenic phenotypes, such as proliferation, migration and invasion^6^. Moreover, many key genes that drive PCa, such as *FOXA1* or *HOXB13* (refs. ^7,8^), promote tumor progression by regulating how AR binds to or activates its target genes. Collectively, these findings highlight the importance of AR biology in this disease.

Multiple phase 3 clinical trials have demonstrated the benefit of AR-targeted therapies on patient survival^9–18^. AR signaling inhibitors (ARSI) are now the standard of care for locally advanced^12^, recurrent^13^, nonmetastatic castration-resistant^9,17,18^, metastatic castration-sensitive^14–16^ and metastatic castration-resistant PCa (mCRPC)^10,11^. However, aggressive PCa frequently escapes these therapies by reactivating AR signaling^19^. Recent analyses of clinical samples have demonstrated that the vast majority of mCRPC samples from patients resistant to ARSI agents abiraterone and enzalutamide exhibit robust AR/nuclear AR staining^20,21^. Therefore, identifying new approaches to target AR is critical to improving survival outcomes for patients with AR-driven mCRPC.

We and other groups have identified tumor alterations that reactivate AR signaling during treatment with an ARSI^2,22–29^. These mechanisms of resistance include AR gene amplification^22,23^, AR enhancer amplification^2,24,25^, AR mutation^26^, AR genomic structural rearrangements^27^, AR splice variants^28^ and polymorphisms in androgen metabolism genes^29^. These alterations invariably result in increased expression or increased activity of the AR protein. Thus, strategies for reducing AR protein levels and AR activity represent a promising approach to overcoming known mechanisms of resistance in ARSI-therapy-resistant AR-driven mCRPC. To identify such strategies, we sought to systematically and quantitatively identify genes that regulate AR protein levels with the goal of identifying next-generation AR-targeting strategies for patients with aggressive AR-driven PCa.

## Results

### Establishment of an endogenous AR mNeonGreen2 fluorescent reporter in PCa cells

**Fig. 1:**
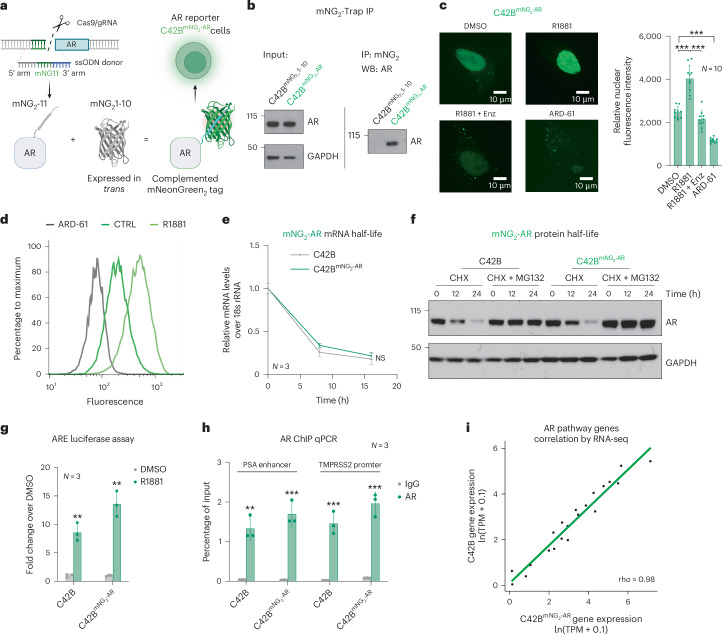
Establishment of an endogenous AR mNG2 fluorescent reporter in PCa cells. **a**, Diagram of the self-assembling split mNG2 fluorescence protein AR tagging approach. A DNA sequence encoding 16-amino-acid 11th β-strand of the fluorescent protein NeonGreen (mNG2_11) is knocked into the N terminus of AR by cotransfection of a homology-directed repair DNA donor and Cas9 RNPs targeting the AR locus. When the remainder of mNG2 (mNG2_1-10) is expressed in *trans* in the same cell, it binds noncovalently to mNG2_11, producing a fluorescent mNG2-AR protein complex. **b**, mNG2 IP followed by western blotting for AR shows formation of mNG2-AR protein complex in *mNG2-AR* endogenously tagged cells but not in controls. **c**, C42B^mNG2-AR^ cells were treated with DMSO, 10 nM R1881 (androgen), 10 nM R1881 plus 10 μM Enz (anti-androgen) or 100 nM ARD-61 (AR degrader, PROTAC) for 24 h. Representative confocal microscopic images showed mNG2-AR localization (green). Nuclear fluorescence intensity was quantified using ImageJ. Ten cells per experimental condition were collected from different fields (*n* = 10; mean ± s.d.). Statistical significance was determined using two-sided *t*-test (***P < 0.001). **d**, C42B^mNG2-AR^ cells were treated with DMSO (CTRL, green), 10 nM R1881 (brighter green) or 100 nM ARD-61 (gray) for 24 h and then mNG2-AR fluorescence was analyzed by flow cytometry. **e**, C42B and C42B^mNG2-AR^ cells were treated with transcription inhibitor 1 μM actinomycin D for 0 h, 8 h or 16 h. RNA was collected to measure AR mRNA levels using real-time PCR (*n* = 3 as biological replicates; mean ± s.d.; NS, no significant difference by two-way ANOVA analysis). **f**, C42B and C42B^mNG2-AR^ cells were treated with protein synthesis inhibitor cycloheximide (CHX, 5 μM) with or without proteasome inhibitor 5 μM MG132 for 0 h, 12 h or 24 h. AR protein levels were detected by western blotting. **g**, C42B and C42B^mNG2-AR^ cells were transfected with androgen response element (ARE)-Luc to enable measurement of AR transcriptional activity. Cells were then treated with DMSO (gray) or 10 nM R1881 (green). ARE-Luc over Renilla was normalized by control (*n* = 3 as biological replicates; mean ± s.d.). Statistical significance was determined using two-sided *t*-test (**P < 0.01). **h**, C42B and C42B^mNG2-AR^ cells were fixed by formaldehyde. ChIP experiments were performed using an IgG (gray) or AR antibody (green). Precipitated DNA fragments were used as templates to amplify the PSA enhancer and TMPRSS2 promoter by real-time PCR (*n* = 3 as biological replicates; mean ± s.d.). Statistical significance was determined using two-sided *t*-test (***P < 0.001; **P < 0.01). **i**, Total RNA from C42B and C42B^mNG2-AR^ cells were collected for RNA-seq. Gene expression values were calculated as ln(TPM + 0.1). Pearson correlation was calculated comparing AR pathway genes^57^ (*n* = 22) between the two cell lines. Panel **a** created with BioRender.com.

**Extended Data Fig. 1:**
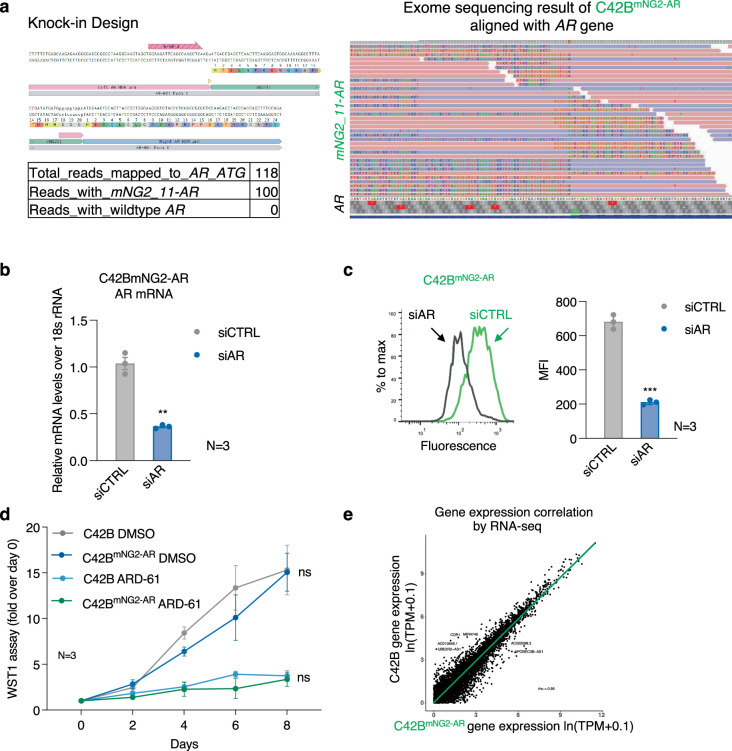
Phenotypical characterization of C42B^mNG2-AR^ cell lines. **a**, Exome sequencing results of C42B^mNG2-AR^ showed a homogenous knock-in of mNG2. Left: *mNG2_11* Knock-in design indicating sequencing location and the reads count from the exosome sequencing. Right: IGV visualization of the sequence alignment around the ATG of AR. **b**, C42B^mNG2-AR^ cells were treated with siRNA targeting control (grey) or AR (blue). RNA was collected, AR mRNA levels over 18 s rRNA were measured by real-time PCR then normalized to siCTRL (n = 3 as biological replicates; Mean ± SD). Statistical significance was determined using two-sided t-test (**P < 0.01). **c**, C42B^mNG2-AR^ cells were treated with siRNA targeting control (green) or AR (black). Cells were collected for flow cytometry, mNG2-AR levels were indicated by the fluorescence intensity. Median Fluorescence Intensity (MFI) was calculated (n = 3; Mean ± SD). Statistical significance was determined using two-sided t-test (***P < 0.001). **d**, C42B (grey) and C42B^mNG2-AR^ cells (blue) were treated with DMSO (solid line) or 50 nM ARD-61 (dashed line). Cell viability was measured by WST1 and normalized over control (n = 3 as biological replicates; Mean ± SD). Statistical significance between C42B and C42B^mNG2-AR^ cells under DMSO or ARD-61 treatment was determined using two-way ANOVA (**P < 0.01; ns=not significant). **e**, Total RNA from C42B and C42B^mNG2-AR^ cells were collected for RNA-seq. Gene expression values were calculated as ln(TPM + 0.1). Pearson correlation was calculated comparing all genes (n = 19127) between the two cell lines. Source data

Accurate reporters of AR activity in PCa cells are essential to developing the next generation of therapeutic approaches that target AR. Existing methods that fuse a full-length fluorescent reporter protein to an endogenous or exogenously overexpressed target protein can affect target function, and are not ideal reporters^30,31^. We previously developed a split fluorescent protein tagging strategy, enabling us to visualize and measure the expression of endogenous genes without antibodies^32^. This approach has advantages, including reduced perturbation to the genomic locus and the protein–protein interactions of the target compared to traditional methods^32–34^. We set out to establish PCa cell line models expressing an endogenous AR split fluorescent reporter fusion protein. To tag AR endogenously, we utilized a two-component split fluorescent protein tagging strategy that breaks the sequence of the monomeric NeonGreen (mNG2) protein between the 10th and the 11th β-strand into two parts: mNG2_1-10 and mNG2_11, with mNG2_11 being a short, 16-amino-acid peptide. Fluorescence arises only when the two protein fragments noncovalently bind, creating a fluorescent protein capable of serving as an endogenous reporter if knocked into the genome in frame to a human gene. We knocked *mNG2_11* into the 5’ protein coding exon of *AR*, in the C42B cell model of aggressive PCa, creating an N-terminally tagged *mNG2_11-AR* fusion gene. We then stably expressed mNG2_1-10 in *trans* using a lentivirus and observed mNG2_1-10 complexes with mNG2_11-AR protein resulting in a bright fluorescent AR protein complex (Fig. 1a–d and Extended Data Fig. 1). We characterized multiple knock-in AR reporter clones (C42B^mNG2-AR^) by extensive genotypic and phenotypic approaches to confirm that tagged AR expression and stability as well as AR biological activity were unperturbed (Fig. 1c–i and Extended Data Fig. 1). Our approach allows us to measure endogenously expressed AR protein rapidly and quantitatively in live or fixed PCa cells, which should enable robust characterization of genetic or chemical perturbations that increase or decrease AR protein levels and localization.

### Identification of regulators of AR using a genome-scale CRISPRi screen

**Fig. 2:**
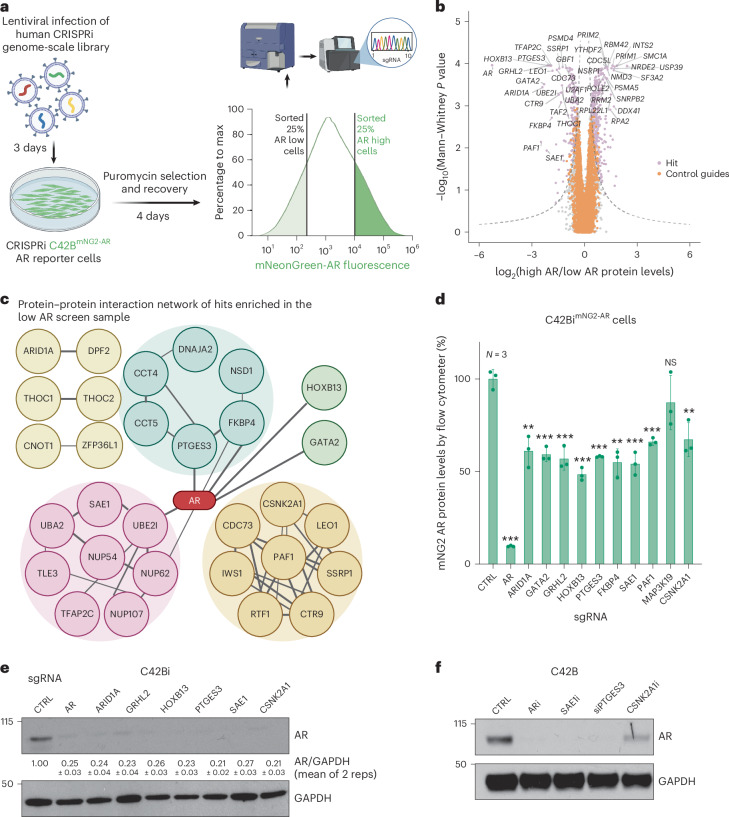
Identification of regulators of AR protein levels using a genome-scale CRISPRi screen. **a**, Schematic of the FACS-based genome-scale CRISPRi screening approach in C42Bi^mNG2-AR^ cells to identify genes that regulate AR protein levels (Methods). **b**, A volcano plot of genome-scale CRISPRi screen hit genes (light purple) that regulate AR protein levels^58^ (Methods). Negative controls are shown in orange. A comparable number of negative control (NC) genes were generated by randomly sampling five nontargeting sgRNAs (with replacement) and analyzed as true genes (*n* = ~19,000). Empirically derived thresholds (log_10_ *P* × phenotype *z* ≥ 13; dashed lines) were calculated as shown, using the NC gene distribution to derive the background s.d. for *z*-scoring. Only a limited number of hits were labeled due to the size of the plot. **c**, A plot showing top positive AR regulator hit (*n* = 51) protein interaction networks analyzed by STRING. Proteins without protein interactions among any of the top 51 hits were removed. Lines between the proteins indicate a confident interaction. Line thickness indicates the strength of data support. Proteins were also unsupervised clustered, indicated by their color. **d**, C42Bi^mNG2-AR^ were infected with sgRNA targeting control or indicated hit genes. After puromycin selection, median fluorescence intensity (MFI) of mNG2-AR fluorescence from the treated cells was measured by flow cytometry. The percentage of AR protein abundance was calculated as AR protein abundance % = (MFI_sg Individual gene _− MFI_C42B_)/(MFI_sgCTRL _− MFI_C42B_) × 100 + 100 (*n* = 3 as biological replicates; mean ± s.d.). Statistical significance was determined using two-sided *t*-test (***P < 0.001; **P < 0.01; NS, not significant). **e**, C42Bi cells were infected with indicated individual sgRNAs targeting hit genes. Cell lysates were collected after puromycin selection. AR and GAPDH levels were detected by western blotting. Densitometry of the replicate western blots (*n* = 2) were calculated by ImageJ and normalized; mean ± s.d. are labeled under the lanes. **f**, C42B cells were treated with 100 nM ARD-61 (ARi), 5 μM ML792 (SAEi), siRNA targeting PTGES3 or 10 μM Slimitasertib (CSNK2A1i) for 24 h. Cell lysates were collected. AR and GAPDH levels were detected by western blotting. Panel **a** and the STRING plot in **c** created with BioRender.com. Source data

**Extended Data Fig. 2:**
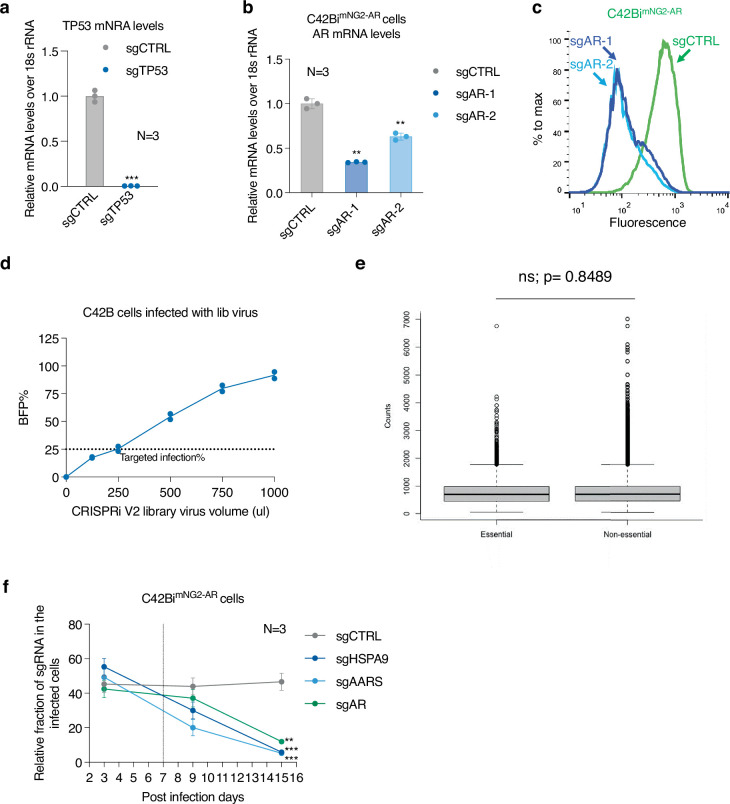
Validation of the CRISPRi activity of C42Bi^mNG2-AR^. **a-b**, C42Bi^mNG2-AR^ cells were infected with sgRNAs targeting CTRL, P53 (**a**) or AR (**b**). RNA was collected; P53 or AR mRNA levels over 18 s rRNA were measured by real-time PCR (n = 3 as biological replicates; Mean ± SD). Statistical significance was determined using two-sided t-test (***P < 0.001, **P < 0.01). **c**, C42Bi^mNG2-AR^ cells were infected with sgRNAs targeting control (green) or AR (blue). Cells were collected for flow cytometry; mNG2-AR levels were indicated by the fluorescence intensity. **d**, C42B cells were infected with increasing amounts of CRISPRi v2 library virus, and BFP-positive percentages were measured by flow cytometry 3 days post-infection to determine the optimal viral dose targeting 25% transduction. Two biological replicates were shown in the plot. **e**, Counts of essential and non-essential sgRNAs at post-infection day 7 (T0) are shown as box-and-whisker plots. The central line denotes the median, the box spans the interquartile range, and open circles represent outliers as defined by the R base boxplot function (values > 1.5× outside the interquartile range). Counts of non-essential sgRNAs (n = 94,070) and essential genes (n = 10,465, defined by DepMap: https://depmap.org/portal/) were compared using a two-sided Mann–Whitney test (P = 0.8489), showing no significant difference between essential and non-essential guides. **f**, A competitive growth-based assay of the C42Bi^mNG2-AR^ cells infected with sgRNA (mCherry + ) targeting CTRL, AR, or commonly essential genes (HSPA9 and AARS) as a positive control. The relative fraction of sgRNA in the cell population was measured by flow cytometer starting from day 3 post-infection. Statistical significance between each targeting sgRNA and sgCTRL were determined using two-way ANOVA (**P < 0.01; ***P < 0.001). Source data

We next set out to use our C42B^mNG2-AR^ cell lines to identify genes that regulate AR protein abundance. We first stably expressed a dCas9-KRAB fusion CRISPRi construct in C42B^mNG2-AR^ cells. Control experiments targeting TP53 and AR demonstrated CRISPRi gene silencing activity (Extended Data Fig. 2a–c). We next performed genome-scale CRISPRi screens in two C42B^mNG2-AR^ clonal cell lines using fluorescence-activated cell sorting to identify genes whose repression would decrease or increase AR protein levels (Fig. 2a and Extended Data Fig. 2d). Genome-scale pooled genetic screens were performed by transducing C42B^mNG2-AR^ stably expressing dCas9-KRAB (C42Bi^mNG2-AR^) with a genome-scale CRISPRi library^35^. Samples were collected, fixed with 3% paraformaldehyde (PFA), and then sorted to isolate cells in the top and bottom quartile of AR fluorescent signal. We extracted genomic DNA and amplified the sgRNAs present and quantified sgRNA abundance in each sample by quantitative sequencing to identify genes that regulate AR levels. We confirmed that our screening strategy could robustly capture phenotypes induced by repression of genes such as AR or other commonly essential genes that induce strong growth phenotypes when repressed using timecourse fluorescent competition growth assays and by analysis of the abundance of commonly essential and nonessential single-guide RNA (sgRNA) in our CRISPRi genome-scale screening data (Extended Data Fig. 2e,f). We chose to focus solely on genes required for maintenance of AR levels, as such genes, if targetable, would be therapeutically relevant to PCa therapy. As expected, sgRNAs targeting AR were enriched strongly in the low AR screen sample, meaning that repression of AR resulted in the greatest decrease in AR protein levels. We also identified other known AR regulators including *HOXB13*, *GRHL2* and *GATA2* as top hits suggesting our screen robustly identifies genes required for AR protein abundance (Fig. 2b). Top hits required for AR abundance include genes involved in chromatin transcription, RNA transportation, transcription factor and post-translation regulation, indicating that numerous biological processes influence AR protein levels. Top gene hits included protein complexes such as PAF and proteins such as HOXB13 and GATA2 known to interact directly or indirectly with AR^36^ (Fig. 2c). Among them, FKBP4, PTGES3, UBE2I, HOXB13 and GATA2 showed evidence for physical subnetwork interactions with AR^37^. Several genes known to be regulated transcriptionally by AR were identified as hit genes, suggesting AR protein levels may be regulated by AR target genes, as has been demonstrated previously for select examples such as FKBP4^38^.

**Extended Data Fig. 3:**
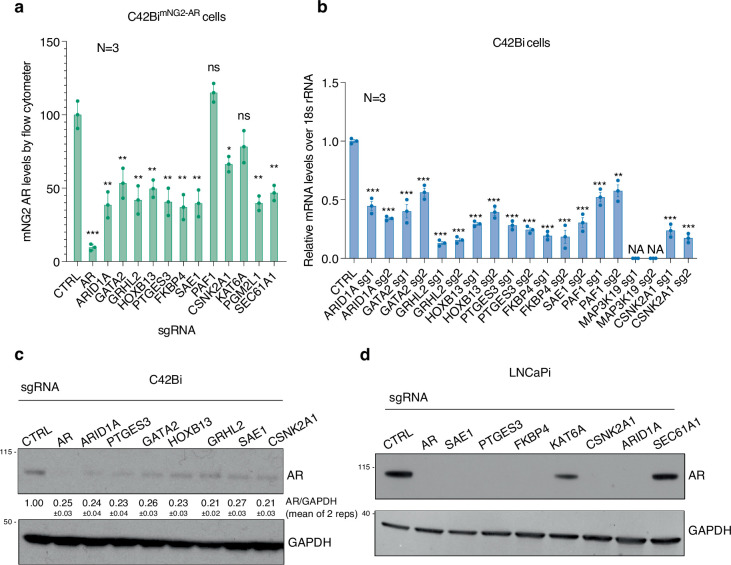
Extended validation of hits from the genome-scale CRISPRi screening. **a**, Clone 2 C42Bi^mNG2-AR^ were infected with sgRNAs targeting control or indicated hit genes. After puromycin selection, Median Fluorescence Intensity (MFI) of mNG2-AR fluorescence from the treated cells was measured by flow cytometry. The percentage of AR expression was calculated as AR expression%=(MFI_sg **Individual gene**_-MFI_C42B_)/(MFI_sgCTRL_-MFI_C42B_)*100 + 100 (n = 3 as biological replicates; Mean ± SD). Statistical significance was determined using two-sided t-test (***P < 0.001; **P < 0.01; ns=not significant). **b**, C42Bi were infected with sgRNAs targeting control or indicated hit genes. mRNA levels of the indicated genes over 18 s rRNA were measured by real-time PCR (n = 3 as biological replicates; Mean ± SD). Statistical significance was determined using two-sided t-test (***P < 0.001; NA=undetectable). **c**, C42Bi cells were infected with indicated individual sgRNAs. Cell lysates were collected after puromycin selection. AR and GAPDH levels were detected by western blotting. Each western blot experiment was performed twice to determine reproducibility. **d**, LNCaPi cells were infected with indicated individual sgRNAs. Cell lysates were collected after puromycin selection. AR and GAPDH levels were detected by western blotting. Each western blot experiment was performed twice to determine reproducibility. Source data

We validated individual top hit genes including *ARID1A*, *GATA2*, *GRHL2*, *HOXB13*, *PTGES3*, *FKBP4*, *SAE1*, *PAF1*, *MAP3K19* and *CSNK2A1* in two C42Bi^mNG2-AR^ clonal cell lines demonstrating our screen results were reproducible and identified genes that modulate AR levels (Fig. 2d and Extended Data Fig. 3a). As a control we confirmed by quantitative PCR (qPCR) with reverse transcription (RT–qPCR) that CRISPRi knockdown of top hits decreased the mRNA expression of each targeted hit gene as expected (Extended Data Fig. 3b). To extend our results beyond the C42Bi^mNG2-AR^ model, we confirmed repression of top hits identified in the screen decreased AR protein levels in parental C42B and LNCaP cells expressing dCas9-KRAB (hereafter C42Bi and LNCaPi), demonstrating the tagged mNG2-AR protein and unmodified AR protein are regulated by the same genes and further confirming the mNG2-AR model as a robust tool for studying AR biology (Fig. 2e and Extended Data Fig. 3c,d). We also validated our screen results using orthogonal genetic and chemical strategies, (ARD-61 for AR, ML-792 for SAE1, siPTGES3 for PTGES3 and CX-4945 for CSNK2A1). In each case, perturbation of candidate genes reduced AR protein levels (Fig. 2f). Our results demonstrate genome-scale fluorescence-activated cell sorting (FACS)-based CRISPRi screens utilizing the C42B^mNG2-AR^ model robustly identified both known and unexpected genes that regulate AR levels.

### PTGES3 is a regulator of AR protein

**Fig. 3:**
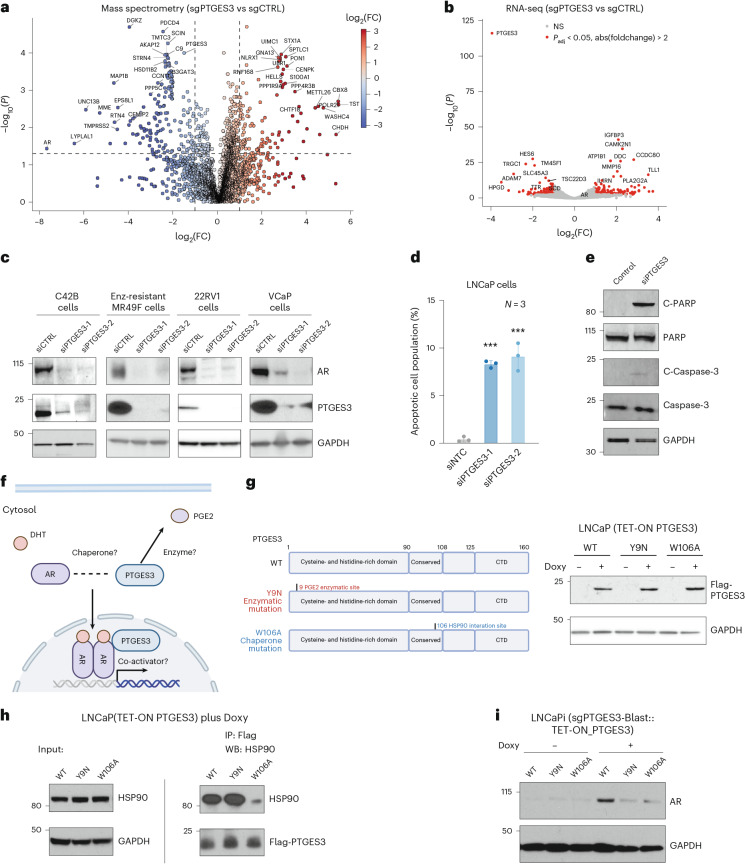
PTGES3 is a regulator of AR. **a**, A plot showing proteomic changes upon repression of PTGES3 relative to controls; 22RV1i cells were infected with sgRNAs targeting CTRL (control) or PTGES3 and then subjected to puromycin selection. Cells were collected for label-free shotgun mass spectrometry (*n* = 3). Volcano plot showing the DE proteins in sgPTGES3 versus sgCTRL groups. **b**, A plot showing gene expression changes upon repression of PTGES3 relative to controls. LNCaPi cells were infected with sgRNAs targeting CTRL (nontargeting control) or PTGES3. Total RNA was collected for RNA-seq (*n* = 2 as biological replicates). DE genes between the two groups were highlighted in red in the volcano plot; top ranking DE genes were labeled. *AR* is not DE when PTGES3 is knocked down. **c**, C42B, Enzalutamide resistant cell lines MR49F, 22RV1 and VCaP were treated with siRNA targeting control or PTGES3. AR, PTGES3 and GAPDH levels were detected by western blotting. **d**, LNCaP cells were treated with siRNA targeting control or PTGES3 for 48 h, then stained with PI/Annexin V. The percentage of the apoptotic population was measured by FACS (*n* = 3 biological replicates; mean ± s.d.). Statistical significance was determined using two-sided *t*-test (***P < 0.001). **e**, LNCaP cells were treated with siRNA targeting control or PTGES3. cleaved PARP, PARP, cleaved Caspase-3, Caspase-3 and GAPDH levels were detected by western blotting. **f**, A diagram showing the potential dual functions of PTGES3 regulating AR. **g**, Left: diagrams illustrating the wildtype, enzymatic mutation (Y9N) and HSP90 mutation (W106A) isoforms of PTGES3. Right: LNCaP cells stably expressing a TET-ON inducible PTGES3 wildtype, Y9N mutation and W106A mutation were treated with or without 100 ng ml^−1^ doxycycline. Flag-PTGES3 and GAPDH levels were detected by western blotting. **h**, LNCaP (TET-ON PTGES3) expressing inducible flag-tagged PTGES3 WT, Y9N mutation or W106A mutation were treated with 100 ng ml^−1^ doxycycline. Co-IP assays were performed using flag antibody. GAPDH, Flag-PTGES3 and HSP90 amounts were detected by western blotting. **i**, LNCaPi cells were infected with sgPTGES3-Blast, then treated with DMSO or 100 ng ml^−1^ doxycycline to overexpress the PTGES3 WT or Y9N or W106A mutant proteins. AR and GAPDH levels were detected by western blotting. Panels **f** and **g** created with BioRender.com. Source data

**Extended Data Fig. 4:**
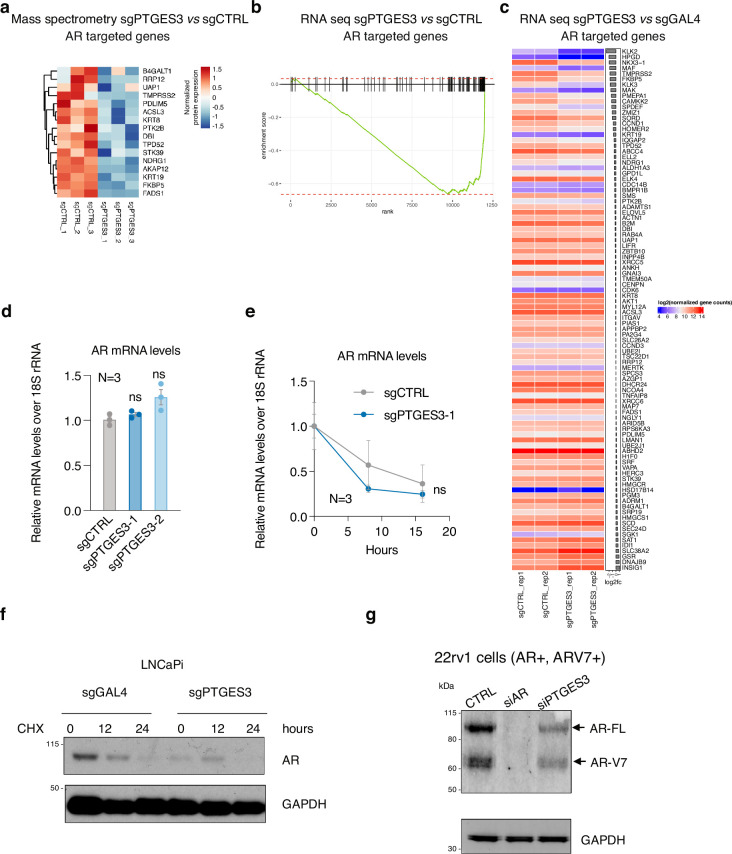
PTGES3 is required for AR protein levels and AR activity but does not affect AR mRNA levels, mRNA half-life or protein half-life. **a**, Analysis of proteomic effects of PTGES3 knockdown versus control for select AR target genes. Mass spectrometry was performed in Fig. 3a and focused results are shown here as a heatmap. **b**, LNCaPi were infected with sgRNA targeting control or PTGES3. Total RNA was collected for RNA-seq. Gene Set Enrichment Analysis (GSEA) was performed. AR targeted genes are the top rank downregulated gene set. **c**, sgPTGES3 vs sgCTRL RNA-seq was performed as described in **b**. The RNA levels of individual Hallmark Androgen response genes were shown in the heatmap. **d**, LNCaPi were infected with sgRNAs targeting CTRL or PTGES3. AR mRNA levels over 18 s rRNA were measured by real-time PCR (n = 3 as biological replicates; Mean ± SD; ns indicates no significant difference by two-sided t-test analysis). **e**. LNCaPi cells infected with sgCTRL or sgPTGES3 were treated with 1uM Actinmycin D for 0, 8 or 16 h. RNA was collected to measure the AR mRNA levels using real-time PCR (n = 3 as biological replicates; Mean ± SEM; ns indicates no significant difference by two-way ANOVA analysis). **f**. LNCaPi cells infected with sgCTRL or sgPTGES3 were treated with protein synthesis inhibitor Cycloheximide (CHX, 5 μM) for 0, 12, 24 h. AR protein levels were detected by western blotting. **g**, 22RV1 cells were treated with siRNA targeting control or PTGES3. AR, ARV7, and GAPDH levels were detected by western blotting. Source data

We were intrigued by the identification of PTGES3 as a top regulator of AR levels. Using unbiased whole cell mass spectrometry-based proteomics analysis, we demonstrated significant downregulation of AR protein upon PTGES3 knockdown, which confirms our western blotting results (Fig. 3a). We also observed downregulation of transcripts and proteins whose expression is associated with AR activity upon PTGES3 knockdown (Fig. 3a,b and Extended Data Fig. 4a–c). Repression of PTGES3 did not alter *AR* mRNA levels as measured by RNA sequencing (RNA-seq) or RT–qPCR, suggesting that PTGES3 regulates AR protein translation or protein stability (Fig. 3b and Extended Data Fig. 4d). Knockdown of PTGES3 does not alter the mRNA or protein half-life of AR (Extended Data Fig. 4e,f). PTGES3 knockdown also decreased levels of AR protein in additional cell models of aggressive mCRPC including 22RV1 cells, AR gene amplified VCaP and ARSI (enzalutamide) resistant MR49F cells (Fig. 3c). PTGES3 knockdown also decreased levels of AR splice variant 7 (V7) in 22RV1 cells that express high levels of both full length and V7 AR protein (Extended Data Fig. 4g). We observed that PTGES3 knockdown increases apoptotic cell populations and induces the expression of cleaved PARP and cleaved caspase-3 (Fig. 3d,e). Collectively, these in vitro preclinical PCa models represent common clinically relevant AR statuses and our data lead us to speculate that a PTGES3 inhibitor could inhibit AR function in diverse high-risk AR-driven mCRPC clinical scenarios.

Having established that PTGES3 regulates AR levels in a variety of aggressive AR-dependent mCRPC cell models, we set out to decipher the mechanism by which PTGES3 supports AR protein levels and thus promotes AR signaling. In the absence of androgen ligands, AR protein is bound by protein chaperones such as HSP90 and localized in the cytosol^39^. Upon androgen stimulation, AR translocates into the nucleus, binds to genomic androgen response elements and modulates gene expression of target genes^40^. As PTGES3 potentially regulates AR in a post-translational manner, we were interested in the protein function as well the localization of PTGES3. PTGES3 protein is reported to have several protein functions and to be localized to both the cytosol and nucleus^41–49^ (Fig. 3f). PTGES3 is reported to have a cytosolic HSP90-dependent protein chaperone function for steroid nuclear receptors, but its activity in PCa is poorly characterized^41,43–46^. PTGES3 is also reported to be a cytosolic prostaglandin synthase that converts Prostaglandin H2 (PGH2) to Prostaglandin E2 (PGE2)^47^; of note, other prostaglandin synthases in the same family as PTGES3 have been implicated in the production of androgens^48,49^. The proposed PGE2 enzymatic site is at the N terminus of the protein whereas the proposed HSP90 interaction motif is at the C terminus of the protein^41,47^.

**Extended Data Fig. 5:**
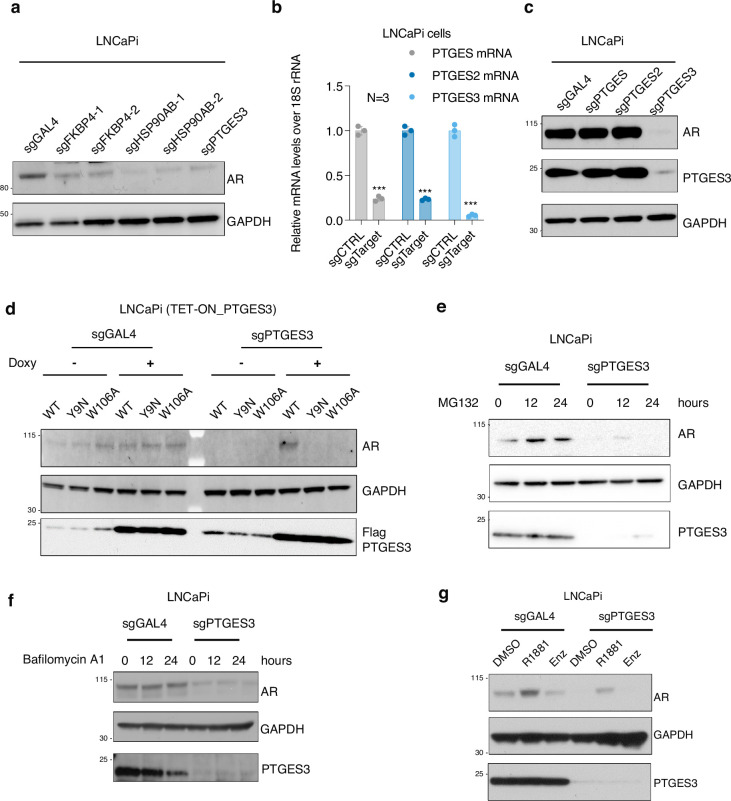
Dual functions of PTGES3 potentially regulating AR protein levels. **a**, LNCaPi were infected with sgRNA targeting CTRL, FKBP4, HSP90 or PTGES3 and then selected with puromycin. Cell lysates were collected. AR and GAPDH levels were detected by western blotting. **b-c**, LNCaPi were infected with sgRNA targeting CTRL, PTGES, PTGES2 or PTGES3 and then selected with puromycin. RNA or cell lysates were collected. Indicated genes mRNA levels over 18 s rRNA were measured by real-time PCR (**b;** n = 3 as biological replicates; Mean ± SD). Statistical significance was determined using two-sided t-test (***P < 0.001). AR, PTGES3 and GAPDH levels were detected by western blotting (**c**). Each western blot experiment was performed twice to determine reproducibility. **d**, LNCaPi cells were infected with sgCTRL-Blast or sgPTGES3-Blast and selected, then treated with DMSO or 100 ng/ml doxycycline to overexpress the PTGES3 WT or Y9N or W106A mutant proteins. AR, PTEGS3 and GAPDH levels were detected by western blotting. **e**, LNCaPi cells infected with sgCTRL or sgPTGES3 were treated with proteasome inhibitor 5 μM MG132 for 0, 12, 24 h. AR, PTGES3 and GAPDH protein levels were detected by western blotting. **f**, LNCaPi cells infected with sgCTRL or sgPTGES3 were treated with lysosomal inhibitor 1 μM Bafilomycin A1 for 0, 12, 24 h. AR, PTGES3 and GAPDH protein levels were detected by western blotting. **g**, LNCaPi cells infected with sgCTRL or sgPTGES3 were treated DMSO, 10 nM R1881 (androgen), or 10 μM Enzalutamide (Enz, anti-androgen). AR, PTGES3 and GAPDH protein levels were detected by western blotting. Source data

To investigate the mechanism by which PTGES3 regulates AR, we confirmed that repression of PTGES3 or known co-chaperones of cytosolic AR, HSP90 or FKBP4, resulted in decreased AR protein levels as expected (Extended Data Fig. 5a). Repression of PTGES3 but not PTGES or PTGES2 reduced total AR protein levels (Extended Data Fig. 5b,c). To dissect which of the two proposed PTGES3 protein functions regulates AR levels, we constructed lentiviral doxycycline-inducible TET-ON C-terminal flag-tagged constructs expressing wildtype PTGES3 (WT), PTGES3 with a proposed PGE2 enzyme activity disrupting mutation^47^ (Y9N) and PTGES3 with a HSP90 interaction site mutation (W106A) (Fig. 3g). We transduced LNCaP (TET-ON PTGES3) with these constructs and created isogenic stable cell lines that overexpressed PTGES3 or PTGES3 mutant proteins upon addition of doxycycline (Doxy) (Fig. 3g). We performed co-immunoprecipitation (co-IP) western blotting experiments on each PTGES3 construct and confirmed that the PTGES3 W106A mutation but not the Y9N mutation abrogates the interaction between PTGES3 and HSP90, indicating that the Y9 site might not interact with HSP90 and that the Y9 site could be critical for HSP90 independent function of PTGES3 (Fig. 3h). To test which of these PTGES3 mutations modulates endogenous AR levels, we knocked down endogenous PTGES3 in LNCaPi and then inducibly overexpressed the PTGES3 WT or W106A or Y9N mutant proteins. As expected, we observed that expression of wildtype PTGES3 rescued AR protein levels. Expression of either the Y9N or W106A mutant PTGES3 protein failed to rescue AR protein levels, suggesting that both sites/functions are required to maintain AR (Fig. 3i and Extended Data Fig. 5d). Furthermore, we observed that loss of AR following PTGES3 knockdown is not rescued by inhibition of either the ubiquitin-proteasome or the lysosomal degradation pathway and is not fully restored in the presence of androgen or enzalutamide (Extended Data Fig. 5e–g).

### Nuclear PTGES3 facilitates AR-mediated transcription

**Fig. 4:**
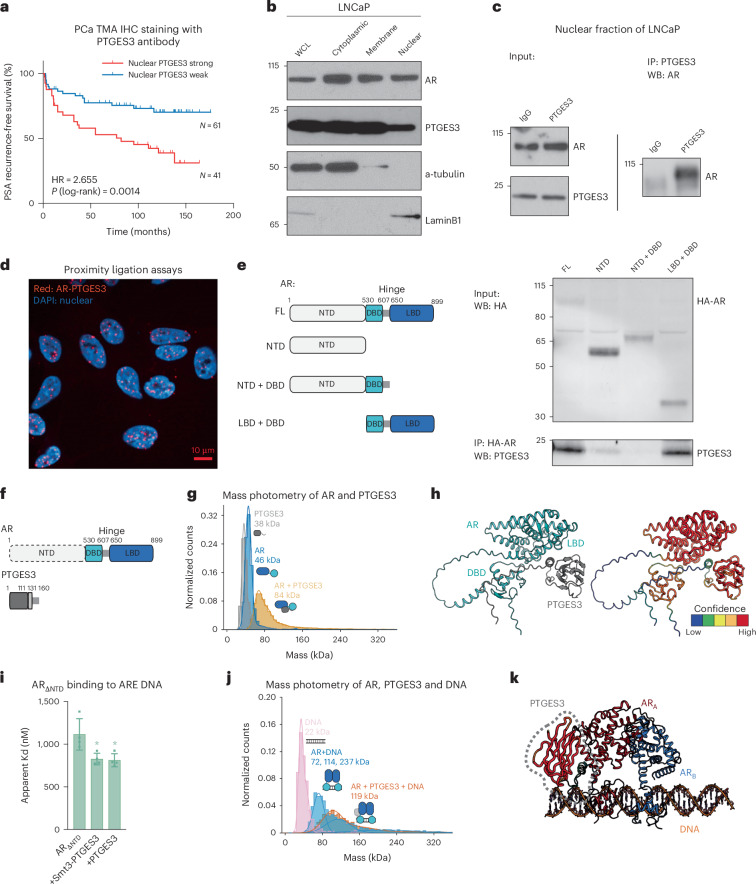
AR and PTGES3 interact in the absence and presence of DNA. **a**, IHC for PTGES3 was performed on human PCa tissue microarrays (*n* = 120). Tissue cores were scored for intensity of nuclear and cytoplasmic PTGES3 staining. Kaplan–Meier graph depicting relation between PSA recurrence-free survival and nuclear PTGES3 staining in PCa patients. Weak, score 0 or 1; Strong, score 2 or 3. **b**, LNCaP cells were fractionated to separate the indicated cellular compartments and then the localization of AR, PTGES3 and control proteins was determined by western blot. **c**, LNCaP cells were fractionated to isolate nuclei and then a co-IP was performed using IgG or PTGES3 antibodies. AR protein levels were detected by western blotting. **d**, A proximity ligation assay was performed and imaged to measure the AR–PTGES3 interaction in intact LNCaP cells. **e**, LNCaP cells were transfection with HA-tagged full-length AR (FL), AR NTD, AR NTD and DBD (NTD + DBD), or AR DBD and LBD (LBD + DBD). Co-IP assays were performed using HA antibody. HA–AR construct protein levels were measured by western blotting of input samples (top). Interactions between HA–AR constructs and PTGES3 were detected by western blotting of Co-IP samples. **f**, Protein domain structures of AR and PTGES3. Disordered domain and regions represented with dashed borders and lines. **g**, AR_ΔNTD_ and Smt3-tagged-PTGES3 interact directly as measured by mass photometry. **h**, AlphaFold modeling of the AR_ΔNTD_ and PTGES3 interaction. **i**, A fluorescence polarization assay measuring binding of AR_ΔNTD_ to fluorescein-labeled double-stranded DNA bearing ARE sequences in the presence and absence of PTGES3. Data presented as mean ± s.d. from *n* = 4 independent experiments, and compared and analyzed to data in Extended Fig. 7f using a two-sided, two-way ANOVA with Tukey’s multiple comparisons test, with AR_ΔNTD_ is the control group (*P < 0.05). +Smt3-PTGES3 versus AR_ΔNTD_, *P* = 0.0254; PTGES3 versus AR_ΔNTD_, *P* = 0.0337. **j**, Mass photometry shows that PTGES3 chaperones AR_ΔNTD_’s binding to DNA (ARE). **k**, Structural modeling of the AR–PTGES3 interaction on DNA-bound AR^50^. Source data

**Extended Data Fig. 6:**
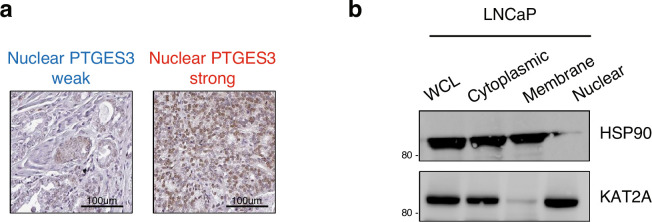
PTGES3 has a nuclear localization. **a**, Representative immunostainings against PTGES3 in PCa patient samples. **b**, Cell fractions from LNCaP cells were immunoblotted with indicated antibodies. Source data

PTGES3 has also been reported to localize to the nucleus and previous publications showed nuclear PTGES3 is a glucocorticoid receptor (GR) co-factor that modulates GR activity in the nucleus at GR response elements^44,45^. We set out to evaluate whether PTGES3 is localized to the nucleus in PCa tumors. We measured PTGES3 localization by immunohistochemistry (IHC) using a validated PTGES3 antibody on a tissue microarray containing 120 PCa tumor biopsies. PTGES3 was present at both cytosolic and nuclear locations with increased PTGES3 protein expression in more advanced/aggressive PCa tumors (Extended Data Fig. 6a). Integrated analysis of PTGES3 localization with clinical outcomes data demonstrated patients with a high nuclear PTGES3 score had worse prostate-specific antigen (PSA)-recurrent free survival, demonstrating that nuclear PTGES3 protein levels are correlated with a more aggressive PCa phenotype (hazard ratio = 2.655, *P* log-rank = 0.0014; Fig. 4a).

We further measured PTGES3 localization in the cytoplasm, membrane and nuclear compartment of LNCaP cells. Western blot analysis demonstrated that PTGES3 was localized to both the cytoplasm and nucleus of LNCaP cells (Fig. 4b). As expected, AR was localized in both the cytoplasm and nucleus whereas HSP90 was localized to the cytoplasm (Fig. 4b and Extended Data Fig. 6b). To investigate whether PTGES3 modulates AR in the nucleus, we first tested whether PTGES3 interacts with AR in the nucleus by co-IP western. Our results demonstrated that PTGES3 interacts physically with AR in the nucleus (Fig. 4c). We then demonstrated by proximity ligation assay that PTGES3 and AR interact in the nucleus of cells (Fig. 4d). AR is composed of an unstructured N-terminal domain (NTD) and central DNA-binding domain and a C-terminal ligand binding domain (LBD) (Fig. 4e). To determine which region of AR binds to PTGES3, we expressed domain mutant *AR* transgenes in cells and then measured the PTGES3/AR interaction by co-IP western blot. We observed that PTGES3 interaction with AR depends on the DBD and LBD (Fig. 4e).

**Extended Data Fig. 7:**
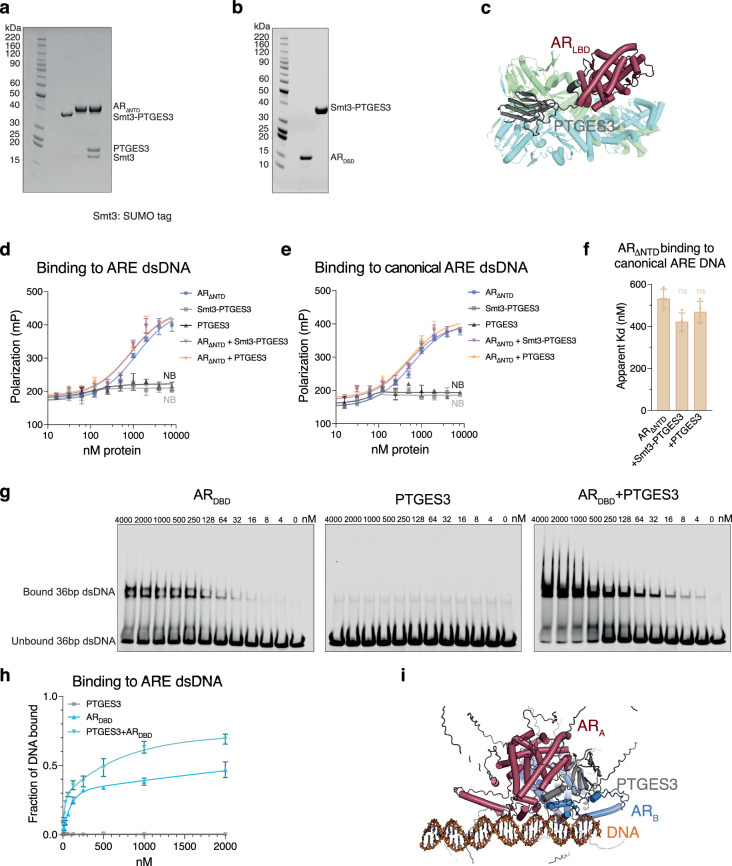
PTGES3 interaction with AR modulates AR’s ability to bind DNA. **a-b**, Protein gels of final recombinant AR and PTGES3 protein preparations used in DNA binding assays. Sypro Ruby-stained 4-12% SDS-PAGE. **c**, Structural modeling of the AR ligand binding domain^67^ (PDB: 1XOW) onto the PTGES3-HSP90 client maturation complex^43^ (PDB: 7KRJ). **d**-**e**, Fluorescence polarization binding curves corresponding to data presenting in Fig. 4i showing AR_ΔNTD_ binding to ARE (**d**) or canonical ARE (**e**) double-stranded DNA. **f**, Fluorescence polarization measuring binding of AR_ΔNTD_ to fluorescein-labeled double-stranded DNA bearing canonical ARE sequences. Data presented as mean ± SD from n = 4 independent experiments, and compared and analyzed to data in Fig. 4i using a two-sided, two-way ANOVA with Tukey’s multiple comparisons test, n.s. (not significant), P > 0.05, where AR_ΔNTD_ is the control group. **g-h**, Electrophoretic mobility shift assays (**g**) and quantitation (**h**) measuring binding of AR_DBD_ and PTGES3 to 36 base pair ARE DNA assayed in **e,f**. Experiments and gels were processed in parallel. Data presented as mean ± SD from n = 3 independent experiments. **i**, AlphaFold modeling of the dimeric AR_FL_ and PTGES3 interaction on ARE double-stranded DNA. Source data

To determine whether AR and PTGES3 directly interact, we performed biochemical reconstitution using recombinant PTGES3 and AR (Extended Data Fig. 7a,b). As we determined that the AR NTD is not implicated in the AR–PTGES3 interaction (Fig. 4e) and because the NTD has been shown to be dispensable for dimerization and DNA binding^50,51^, we assayed AR variants lacking this disordered domain (AR_ΔNTD_ and AR_DBD_). We first demonstrated that PTGES3 and AR_ΔNTD_ directly interact by mass photometry (Fig. 4f,g), which is supported by structural modeling of cytosolic complexes where PTGES3 is predicted to interact with the AR LBD in the absence and presence of the chaperone HSP90 (Fig. 4h and Extended Data Fig. 7c). As we observed PTGES3-AR interactions in the nucleus (Fig. 4c,d), we next queried whether PTGES3 alters the ability of AR to interact with DNA. We measured that PTGES3 promoted the ability of AR to bind double-stranded DNA bearing androgen response element (ARE) sequences, despite PTGES3 having no intrinsic DNA-binding activity (Fig. 4i and Extended Data Fig. 7d–f). PTGES3 addition appears to chaperone DNA binding of AR, restricting formation of heterogenous complexes^50^ as observed by mass photometry (Fig. 4j). Productive PTGES3 interaction with DNA-bound AR is supported by structural modeling, where PTGES3 does not clash with known AR allosteric or DNA-binding interactions, and is wedged between both the AR LBD and DBD, seemingly reinforcing these contacts (Fig. 4k and Extended Data Fig. 7i). In support of these structural models, we demonstrated that PTGES3 can also augment DNA binding of the AR DBD (Extended Data Fig. 7g,h), consistent with our observation that PTGES3 promotes stabilization of AR splice variant 7 (Extended Data Fig. 4g). Taken together, these findings demonstrate that PTGES3 and AR interact in cytoplasmic and nuclear compartments in cells, and that these interactions can be reconstituted in vitro and shown to alter the ability of AR to bind ARE DNA.

**Fig. 5:**
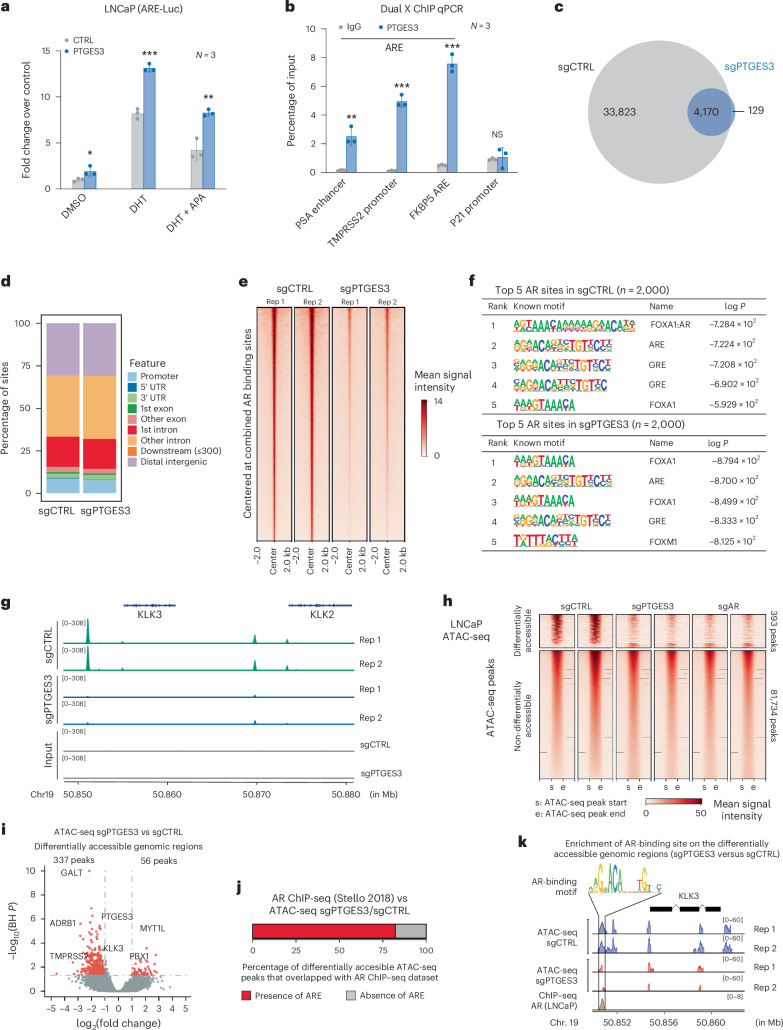
Nuclear PTGES3 facilitates AR-mediated transcription. **a**, LNCaP cells containing ARE–luciferase reporter were transfected with pCDH-control vector (gray) or pCDH-PTGES3 (blue). Cells were treated with DMSO, 1 nM DHT or 1 nM DHT plus 5 μM apalutamide (APA). Luciferase activities over Renilla were normalized with control (*n* = 3 as biological replicates; mean ± s.d.). Statistical significance was determined using two-sided *t*-test (DMSO group *P = 0.0435; DHT group ***P = 0.0002; DHT + APA group **P = 0.0040). **b**, LNCaP cells were fixed sequentially by EGS and formaldehyde. Dual crosslinking ChIP experiments were performed using indicated antibodies. Precipitated DNA was used as a template to amplify the indicated genomic regions by real-time PCR (*n* = 3 as biological replicates; mean ± s.d.). Statistical significance was determined using two-sided *t*-test (**P < 0.01; ***P < 0.001; NS, not significant). **c**, Venn diagram showing overlaps of AR ChIP–seq peaks in LNCaP(sgCTRL) and LNCaP(sgPTGES3). **d**, Peak distribution of ChIP-AR peaks in LNCaP(sgCTRL) and LNCaP(sgPTGES3) across different genomic locations. **e**, ChIP-AR read-density heatmaps of LNCaP(sgCTRL) and LNCaP(sgPTGES3) centered at combined ChIP-AR peaks (*n* = 38,122). **f**, The five most enriched known HOMER motifs in the top 2,000 significant ChIP-AR peaks in LNCaP(sgCTRL) and LNCaP(sgPTGES3). **g**, An IGV screenshot of ChIP-AR enrichment in LNCaP(sgCTRL) and LNCaP(sgPTGES3) at enhancer and promoter sites of KLK3 and KLK2 genes. **h**, LNCaPi cells were infected with sgCTRL, sgPTGES3 or sgAR. Cells were collected for ATAC-seq (*n* = 2 as biological replicates). The heatmap shows the differentially accessible ATAC-seq peak regions in sgPTGES3 or sgAR as compared to that in sgCTRL. Each ATAC-seq peak region was scaled to the same size from the start to the end region. Flanking ±500 bp regions are also shown. The top panel shows the corresponding ATAC-seq signal intensity. **i**, The volcano plot shows the differentially accessible ATAC-seq peak regions upon PTGES3 knockdown as compared to controls. Each dot represents an individual ATAC-seq peak. Differentially accessible ATAC-seq peaks are highlighted in red color (*P* < 0.05 and log2 Foldchange >1 or <−1). The nearest gene to several representative differentially accessible ATAC-seq peaks have been highlighted. **j**, A graph depicting the overlap in genomic loci with differentially accessible ATAC-seq peaks upon PTGES3 knockdown that are or are not also bound by AR as measured by ChIP–seq in LNCaP cells^52^ (Stello2018). The figure shows the percentage of these differentially accessible ATAC-seq peaks that overlapped with the AR ChIP–seq dataset. **k**, Plots showing ATAC-seq peak coverage in the KLK3 gene body and flanking ±5 kb region. sgCTRL ATAC-seq peaks are colored in blue and sgPTGES3 ATAC-seq peaks in red. The upstream promoter region of KLK3 contains overlapping ATAC-seq peaks and AR Chip–seq peaks^52^. AR binding motif was identified in this region.

**Extended Data Fig. 8:**
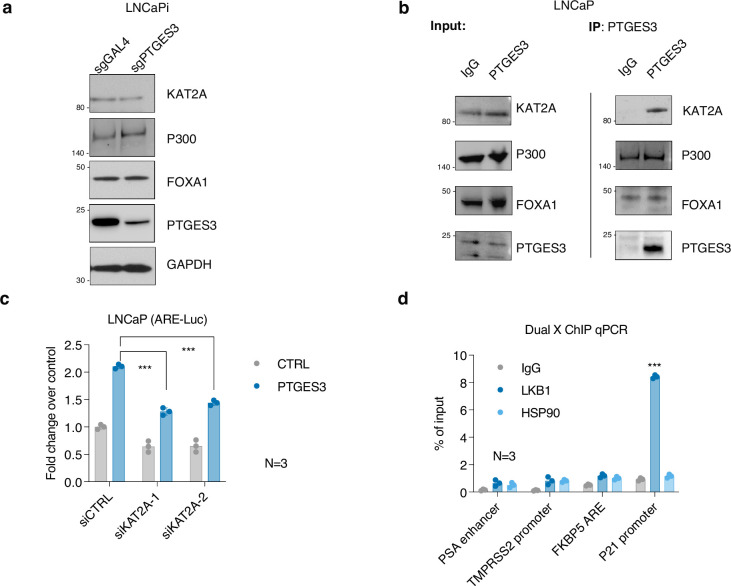
PTGES3 potentially interacts with histone acetyltransferase KAT2A. **a**, LNCaPi cells were infected with sgCTRL or sgPTGES3 and selected with puromycin. KAT2A, P300, FOXA1, PTGES3, and GAPDH protein levels were detected by western blotting. **b**, IP experiments were performed with LNCaP cell lysate using an IgG or PTGES3 antibody. KAT2A, P300, FOXA1, and PTGES3 protein levels were detected by western blotting. **c**, LNCaP cells containing ARE-luciferase reporter were transfected with pCDH-PTGES3. Cells were treated with siRNA targeting control or KAT2A in the condition of 1 nM DHT. Luciferase activities over Renilla were normalized with control (n = 3 as biological replicates; Mean ± SD). Two sided t-test was used to determine statistical significance (***P < 0.001). **d**. LNCaP cells were fixed sequentially by EGS and formaldehyde. Dual cross-linking ChIP experiments were performed using indicated antibodies. Precipitated DNA was used as a template to amplify the indicated genomic regions by real-time PCR (n = 3 as biological replicates; Mean ± SD). LKB1 serves as a positive Dual X ChIP control which indirectly binds to the P21 promoter (non-ARE) by interacting with P53. The P21 promoter levels in LKB1 group were compared to the IgG group, two sided t-test was used to determine statistical significance (***P < 0.001). Source data

We next wanted to test whether PTGES3 promotes AR transcriptional activity and binding to DNA containing AREs in cells. Overexpression of PTGES3 increased AR activity significantly as measured by an ARE–luciferase transcriptional response reporter activity assay, further supporting the hypothesis that PTGES3 expression promotes AR activity (Fig. 5a). We observed repression of PTGES3 does not perturb the abundance of p300, KAT2A or FOXA1, which are transcriptional regulators of AR activity (Extended Data Fig. 8a). Consistent with a previous report^44^, among the transcriptional regulators p300, KAT2A and FOXA1, nuclear PTGES3 interacts with histone acetyltransferase KAT2A by co-IP western (Extended Data Fig. 8b). Knocking down KAT2A inhibited the enhanced AR activity observed upon overexpression of PTGES3 (Extended Data Fig. 8c). This evidence demonstrates that PTGES3 potentially facilitates interactions with AR and transcriptional machinery in the nucleus.

**Extended Data Fig. 9:**
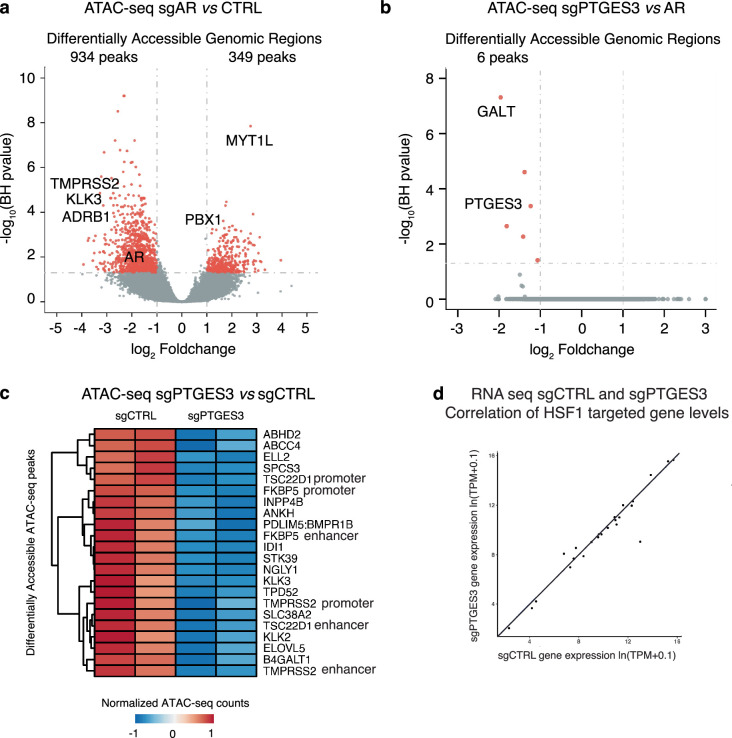
PTGES3 promotes the chromatin opening status of AR binding to AREs. **a**, LNCaPi cells were infected with sgCTRL or sgAR and selected with puromycin. Cells were collected for ATAC-Sequencing. The volcano plot shows the differentially accessible ATAC-seq peak regions in sgAR as compared to that in sgCTRL. Each dot represents an individual ATAC-seq peak. Differentially accessible ATAC-seq peaks are highlighted in red color (pvalue < 0.05 and log2 Foldchange > 1 or <-1). Nearest gene to several representative differentially accessible ATAC-seq peaks have been highlighted. **b**, The volcano plot shows the differentially accessible ATAC-seq peak regions in sgPTGES3 as compared to that in sgAR. **c**, The differentially accessible ATAC-seq peaks (in sgPTGES3 as compared to that in sgCTRL) were mapped to the promoter or enhancer regions. The nearest genes to the regions were selected. AR target genes from the two groups were shown in the heat map. **d**, Total RNA from LNCaP(sgCTRL) and LNCaP(sgPTGES3) cells were collected for RNA-seq. Gene expression values were calculated as ln(TPM + 0.1). Pearson correlation was calculated comparing expression levels of HSF1 target genes between the two cell lines. Source data

To test the hypothesis that a PTGES3/AR protein complex is localized to canonical endogenous AREs and AR target genes in cells, we assessed PTGES3 genomic localization by indirect protein–DNA interactions by dual crosslinked immunoprecipitation (IP) followed by real-time PCR for DNA sequences of interest (Dual × chromatin immunoprecipitation (ChIP) sequencing (ChIP–seq)). Our results demonstrated that PTGES3 is specifically enriched at AREs that are hallmark AR targets but not in non-AR binding region or control regions (Fig. 5b). We observed that HSP90 is not enriched at hallmark AREs, further supporting the hypothesis that nuclear PTGES3 plays an HSP90-independent role in promoting AR activity (Extended Data Fig. 8d). We next used ChIP–seq to examine AR occupancy across the genome in the presence or absence of PTGES3. We observed loss of AR binding at more than 85% of AR binding sites across the genome upon PTGES3 knockdown, including a near complete loss of AR binding at hallmark AREs such as KLK3 (Fig. 5c–g). Loss of AR binding occurred broadly without trends for specific genomic annotations such as promoters, introns or intergenic regions (Fig. 5d). We observed that AR binding annotations in the presence and absence of PTGES3 were similar suggesting loss of PTGES3 reduces AR binding across the genome but does not induce an unexpected AR cistrome (Fig. 5f). To test whether PTGES3 activity promotes chromatin accessibility and therefore potentially AR binding to regulatory elements, we performed assay for transposase-accessible chromatin using sequencing (ATAC-seq) comparing PTGES3 knockdown or AR knockdown to controls or to each other (Fig. 5h). We observed repression of PTGES3 results in far more loss of open chromatin regions (OCRs) than gain of OCRs, consistent with observations from AR knockdown (Fig. 5i and Extended Data Fig. 9a). We also compared the differentially accessible regions observed upon knockdown of AR or PTGES3 to each other. We observed the changes in chromatin accessibility were highly similar, indicating repression of AR and PTGES3 phenocopy each other (Extended Data Fig. 9b). We then mapped the differentially accessible DNA regions to the nearest gene promoter and observed a strong enrichment for genes that are hallmark androgen response genes (Extended Data Fig. 9c). Integration of our ATAC-seq data with AR ChIP–seq data^52^ demonstrated that more than 80% of the differentially accessible ATAC-seq peaks overlapped with AR ChIP–seq peaks (Fig. 5j). For example, knockdown of PTGES3 significantly reduced the chromatin accessibility at the canonical ARE of the *KLK3* promoter (Fig. 5k). These data support the hypothesis that nuclear PTGES3 forms a protein complex with AR that is required for AR protein stability and transcriptional activity in AR-driven PCa. Furthermore, our results suggest PTGES3 biology is distinguished from cytoplasmic AR co-chaperones such as HSP90, as PTGES3 knockdown does not alter expression of HSF1 target genes linked to the HSP90 pathway (Extended Data Fig. 9d), supporting the notion that an anti-cancer strategy targeting PTGES3 will not phenocopy targeting HSP90.

### PTGES3 is a potential therapeutic target for AR-directed therapy resistant PCa

**Fig. 6:**
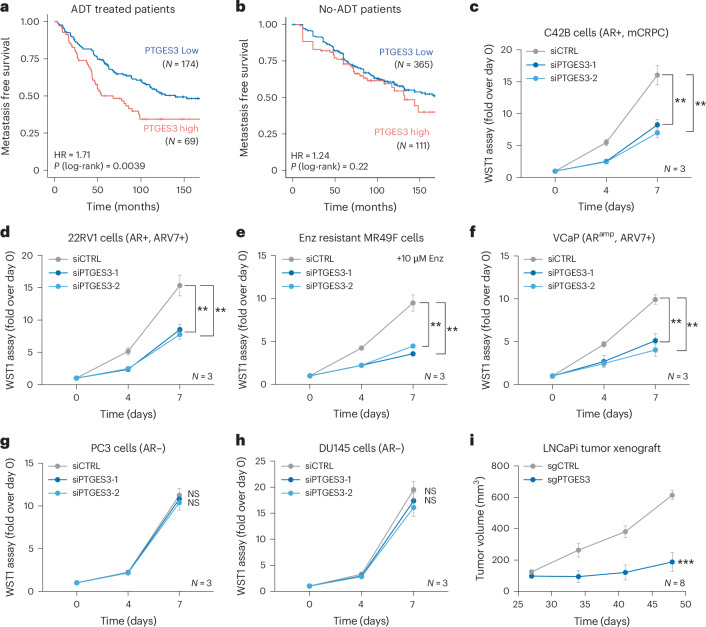
PTGES3 is a potential therapeutic target for AR-directed therapy resistant PCa. **a**,**b**, In a previously established cohort matched for eight clinicopathologic prognostic variables^59^, high PTGES3 expression was associated with worse MFS in prostatectomy patients who received adjuvant ADT (HR:1.71, log-rank *P* = 0.0039) (**a**), but not in prostatectomy patients who did not receive adjuvant ADT (HR:1.24, log-rank *P* = 0.22) (**b**). **c**–**h**, C42B (**c**), 22RV1 (**d**), Enzalutamide (Enz) resistant cell line MR49F cultured with 5 μM Enz (**e**), VCaP (**f**), PC3 (**g**) or DU145 (**h**) cells were treated with siRNA targeting control (gray) or PTGES3 (blue). Cell viability was measured by WST1 and normalized over control (*n* = 3 as biological replicates; mean ± s.d.). Statistical significance of siPTGES3-1 versus siControl (compare 1) and siPTGES3-2 versus siControl (compare 2) were determined using two-way ANOVA. In C42B cells (**c**), 1 **P = 0.0016 and 2 **P = 0.0019; in 22rv1 cells (**d**), 1 **P = 0.0016 and 2 **P = 0.00196; in MR49F cells (**e**), 1 **P = 0.0016 and 2 **P = 0.001; in VCaP cells (**f**), 1 *P* = 0.0015 and 2 **P = 0.0013; in PC3 cells (**g**), 1 P = 0.4228 (NS) and 2 *P* = 0.2013 (NS) and in DU145 cells (**h**), 1 *P* = 0.1017 (NS) and 2 P = 0.05265 (NS). **i**, Mice bearing LNCaP(TET-ON dCas9-KRAB) stably expressing CTRL or PTGES3 sgRNAs were treated with doxycycline when tumors reached ~200 mm^3^. Average tumor volume was plotted (*n* = 8; ± s.e.m.) mean and two-way ANOVA was used to measure statistical significance (***P = 0.0002). Source data

**Extended Data Fig. 10:**
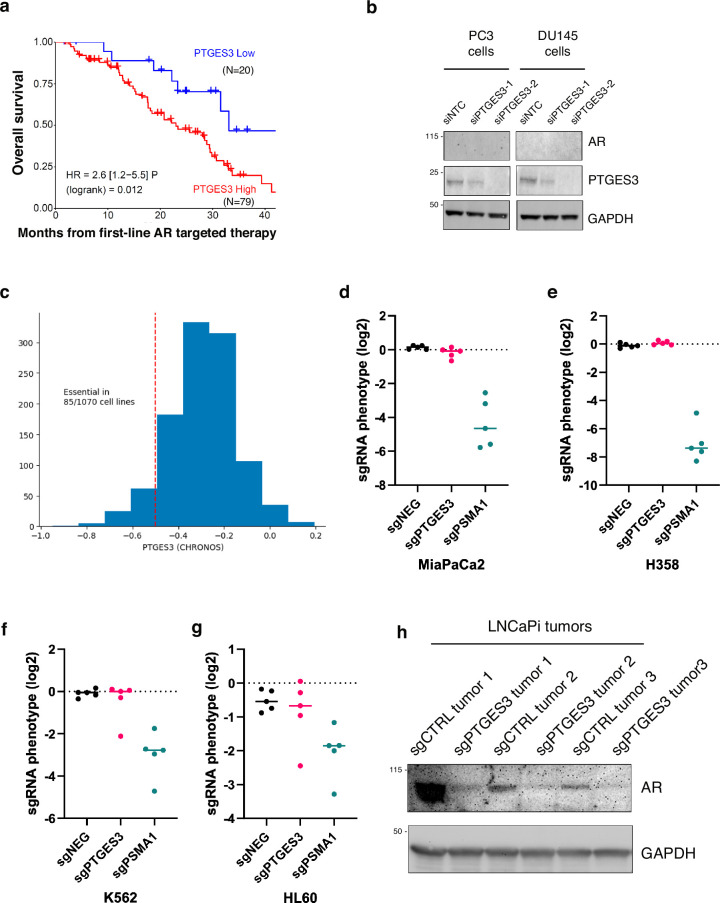
High PTGES3 expression predicts a poor outcome in advanced PCa patients treated with first line AR targeted therapy. **a**, Kaplan-Meier analysis of mCRPC patients treated with a first-line AR targeted therapy (abiraterone acetate, enzalutamide, or apalutamide) shows a worse overall survival in patients with high PTGES3 expression compared to low expression. **b**, PC3 or DU145 cells were treated with siRNA targeting control. were collected for western blot using AR, PTGES3, and GAPDH antibodies. **c**, A histogram of pan-cancer essentiality CHRONOS scores from the Cancer Dependency map showing the distribution of PTGES3 essentiality in 1070 cell lines. PTGES3 is considered essential for cell proliferation or survival in 85 cell lines ( ~ 10% of all cell lines) with scores less than -0.5. This result indicates PTGES3 is not required for the survival or growth of the vast majority of non-prostate cancer or AR independent cell lines. **d-g**, CRISPRi sgRNA level screen phenotypes performed in MiaPaCa2(**c**), H358(**d**), K562(**e**), and HL60(**f**) cells were plotted as log2 values for PTGES3, for PSMA1 which is a common essential gene and for 5 randomly selected non-targeting sgRNA controls present in the sgRNA library. **h**, Tumor tissues from Fig. 4i were collected for western blot using AR and GAPDH antibodies. Source data

We next asked whether *PTGES3* expression is associated with PCa disease progression in mCRPC patient cohorts. We utilized a clinical-grade Affymetrix Human-Exon microarray to determine *PTGES3* expression levels in 641 prostatectomy samples from patients with high-risk PCa. When we matched patients by clinicopathologic variables, including Gleason score, PSA, tumor stage and nodal status, we determined that *PTGES3* overexpression was associated with significantly worse metastasis-free survival (MFS) in patients who received adjuvant first-line androgen deprivation therapy (ADT) (with leuprolide or an GnRH agonist) after prostatectomy, but not in patients who did not receive adjuvant ADT after prostatectomy (Fig. 6a,b). Furthermore, higher tumor expression of *PTGES3* was correlated with worse overall survival in mCRPC patients treated with a first-line AR-targeted therapy (abiraterone, enzalutamide or apalutamide) (Extended Data Fig. 10a).

With these clinical biomarker results, we further evaluated PTGES3 as a therapeutic target in several models of aggressive PCa. We knocked down PTGES3 in multiple AR-dependent and AR-independent PCa cell lines and measured cell survival and proliferation. We found PTGES3 is required for cell proliferation in multiple AR-driven PCa models (C42B, 22RV1, VCaP and MR49F cells) but not AR-independent PCa models (PC3 and DU145 cells) (Fig. 6c–h and Extended Data Fig. 10b). Notably, analysis of PTGES3 gene essentiality upon Cas9 knockout across 1,070 diverse cancer cell lines present in the Cancer Dependency Map^53^ demonstrated that PTGES3 is not classified as a commonly essential gene (Extended Data Fig. 10c). Furthermore, CRISPRi experiments in other cancer cell types (AML, CML, pancreatic cancer and lung cancer cell lines^54–56^) demonstrated that, unlike PSMA1, which is a positive control commonly essential gene, PTGES3 is not an essential gene upon knockdown (Extended Data Fig. 10d–g). To test whether PTGES3 is required for AR-dependent PCa cell growth or survival in vivo, we transduced an inducible CRISPRi LNCaP cell model (TET-ON dCas9-KRAB) with sgRNAs targeting PTGES3 or controls and transplanted cells into mice. At tumor onset we administered doxycycline to the mice to induce PTGES3 repression and tracked tumor size over time. We observed that repression of PTGES3 significantly delayed tumor growth in vivo relative to controls (Fig. 6i). Analysis of AR protein levels in tumors demonstrated PTGES3 repression resulted in decreased AR protein abundance in vivo (Extended Data Fig. 10h). Together, these results demonstrate that PTGES3 is required for AR levels and activity, is conditionally essential in diverse AR-driven PCa models but not in non-AR-driven cancer cells and is associated with poor prognosis in PCa patients

## Discussion

We have developed an AR endogenous reporter and used this resource together with genome-scale CRISPRi screens to systematically identify networks of genes that sustain AR protein levels and thus the oncogenic activity of AR in PCa. Our results point to unexpected biological activities that promote AR biology in aggressive PCa. Specifically, we determined that PTGES3 affects AR levels and the activity of AR in the nucleus through a direct interaction with the AR LBD and DBD. Our results reveal that the PTGES3/AR interaction is required for mCRPC cell proliferation and viability in vitro and in vivo, including in models of patients with intrinsic and acquired AR-directed therapy resistance. These findings emphasize the critical role of PTGES3 in the regulation of AR signaling and highlight its potential as a therapeutic target. Future therapeutic strategies targeting PTGES3 could be transformative for treating patients with AR-driven metastatic PCa, especially those resistant to current US Food and Drug Administration-approved ARSIs. By targeting PTGES3, we may be able to overcome the significant challenge of drug resistance, addressing a principal unmet medical need and ultimately improving survival outcomes for patients suffering from this lethal disease.

## Methods

### Ethical statement

All experiments detailed in this paper were performed in compliance with the Institutional Review Board and the Institutional Animal Care and Use Committee (IACUC) at the University of California, San Francisco and Fred Hutchinson Cancer Center. All animal studies were conducted in compliance with UCSF IACUC guidelines with protocol number AN182067.

### Cell lines and reagents

Cell lines LNCaP, C42B, 22RV1, VCaP, DU145, PC3 and 293T were purchased from the American Type Culture Collection (ATCC) and cultured per ATCC protocols in RPMI 1640 (Gibco) or DMEM (ATCC) with 10% FBS (Gibco) in a humidified 5% CO_2_ incubator at 37 °C. MR49F cells were a gift from A. Zoubeidi (Vancouver Prostate Center) and maintained in RPMI 1640 with 10% FBS containing 10 μM Enzalutamide. Cell line short tandem repeat authentications were conducted (University of California (UC) Berkeley DNA Sequencing facility). All chemicals, unless otherwise stated, were purchased from Sigma Aldrich, Enamine, Combi-blocks or Astatech. Enzalutamide and Apalutamide were purchased from Selleckchem. ML-792 was purchased from MedKoo. AR PROTAC degrader ARD-61 was a gift from S. Wang (University of Michigan).

### Endogenous AR reporter cell C42B^mNG2-AR^

Synthetic sgRNAs (Supplementary Table 1) and Cas9 2NLS nuclease were purchased from Synthego. For the knock-in of mNG2_11, 200-nt homology-directed recombination templates were ordered in single-stranded DNA form as ultramer oligos (Integrated DNA Technologies). C42B cells were treated with 50 ng ml^−1^ nocodazole (Sigma) for 14 h before electroporation. Cas9–sgRNA RNP complexes were assembled with 100 pmol Cas9 protein and 130 pmol sgRNA before electroporation and combined with 375 pmol homology-directed recombination template and 1 µg pCE-mp53DD (Addgene, cat. no. 41856) in a final volume of 25 µl. Electroporation was carried out following the protocol of SF Cell Line 4D-Nucleofector X Kit (Lonza, cat. no. V4XC-2024). Nocodazole-treated cells (1 × 10^6^) were collected and resuspended to 75 µl SF solution. RNP/template/pCE-mp53DD mix (25 μl) was then added to cell suspension. Cells were electroporated immediately with 4D-Nucleofector (Lonza) program EN-120 and transferred to a six-well plate with prewarmed medium. Electroporated cells were recovered for 5 days before infection with lentivirus expressing mNG2_1-10 (Addgene, cat. no. 82610). At 72 h after infection, mNG2 fluorescence positive cells were sorted by flow cytometry into single clones. Genomic DNA from AR reporter single clones were extracted for sanger sequencing (MCLab) as well as exome sequencing (QB3 UC Berkeley) to verify the mNG2-11 insertion.

### CRISPRi flow cytometry screen

The genome-scale CRISPRi screens were performed following the Weissman laboratory protocol (weissmanlab.ucsf.edu) as we reported previously^60^. In brief, two individual clones of C42B^mNG2-AR^ cells (clone 1 and clone 2) were infected with a lentivirus encoding the CRISPRi dCas9-BFP-KRAB protein (Addgene, cat. no. 46910). Following infection, CRISPRi BFP^+^ cells were sorted to purity, expanded and CRISPRi silencing activity was confirmed. These cells which stably express CRISPRi were used for both the genome-scale screens and subsequent experiments^58^. The genome-scale CRISPRi-V2 library (Addgene cat. no. 1000000091) virus was generated and titered following the large-scale lentivirus production protocol (https://weissman.wi.mit.edu/resources/) (Extended Data Fig. 3f). Approximately 150 million C42Bi^mNG2-AR^ cells were then infected at 30% infection in duplicate with the CRISPRi-V2 lentiviral library with 8 µg ml^−1^ polybrene (TR-1003-G), to achieve an average of 500× cells per library sgRNA after transduction. After 3 days, cells were selected using 8 µg ml^−1^ puromycin (−03) for 3 days and then allowed to recover without puromycin for 24 h. Cells were then harvested and fixed in 3% PFA (5 million cells per 1 ml of PFA solution) at room temperature for 20 min, washed with cold 1× phosphate-buffered saline (PBS), quenched by 30 mM glycine/PBS (pH 7.5), washed and resuspended in cold FACS buffer (PBS + 10% FBS) and sorted based on mNG2-AR expression levels on a BD FACSAria Fusion cell sorter to collect the 25% highest and 25% lowest cells. Genomic DNA was harvested with the QIAamp DNA FFPE Tissue Kit (cat. no. 56404). A standard genomic DNA PCR (22 cycles) was performed using NEB Next Ultra II Q5 master mix and primers containing TruSeq Indexes for next-generation sequencing (NGS) analysis. NGS libraries were sequenced on a HiSeq 4000. The genome-scale CRISPRi screens were performed in two independent C42Bi^mNG2-AR^ clones. Clone 1 was tested in a single replicate as a pilot screen experiment, while Clone 2 was tested in two screen replicates. Common hits identified in both clones were nominated for follow-up.

### CRISPR screen data analysis

Raw sequencing data were processed using the Weissman laboratory ScreenProcessing^35^ protocol and GitHub pipeline (https://github.com/mhorlbeck/ScreenProcessing). After setting up the Python environment and required library files as outlined in the tutorial document, sgRNA counts were generated from the raw sequencing files using the command: ‘run fastqgz_to_counts.py -p 6–trim-start 1_end 29 library_reference/CRISPRi_V2_human.trim_1_29.’ To calculate sgRNA-level and gene-level phenotypes and p-values, the process_experiments.py function was used. The corresponding configuration files are available at GitHub (https://github.com/haolongli87/ptges3_resources). Raw sgRNA counts and phenotype scores are provided in Source Data Fig. 2. To validate the nominated hits, gene-level phenotype scores were also calculated using the MAGeCK pipeline^61^ based on sgRNA raw counts from both clones, and results are provided in Source Data Fig. 2.

### Plasmids, luciferase reporter assays and siRNAs

PTGES3 ORF (Addgene, cat. no. 108224) was cloned into pLVX-TetOne-Puro-C-Flag vector using Gateway cloning (Invitrogen). PTGES3 mutant constructs were cloned with Q5 Site-Directed Mutagenesis (NEB). Guide RNAs expression vector targeting CTRL or individual genes were cloned into pU6-sgRNA EF1Alpha-puro-T2A-mCherry or Lenti-sgRNA-blast (Addgene, cat. no. 104993). Cloning primers sequences were listed in Supplementary Tables 2 and 3. HA-tagged AR construct plasmids were obtained from Addgene, HA-FL-AR (Addgene, cat. no. 171234), HA-AR-NTD (Addgene, cat. no. 171235), HA-AR-NTD-DBD (Addgene, cat. no. 171236), HA-AR-DBD-LBD (Addgene, cat. no. 171236). The ARE was cloned from ARR3tk plasmid (Addgene, cat. no. 132360) into pGL4 vector (Promega) to construct the ARE–luciferase reporter (ARE-luc). Luciferase activities were determined using the luciferin reagent (Promega) according to the manufacturer’s protocol. Transfection efficiency was normalized to Renilla luciferase activities. For siRNA knockdown experiments, cells were transfected with siRNA against control (siNTC, Dharmacon, cat. no. D-001810-10-05), PTGSE3 (siPTGES3-1 Dharmacon, cat. no. J-004496-10-0002, siPTGES3 Dharmacon, cat. no. J-004496-10-0002), or AR (siAR-1 Dharmacon, cat. no. J-003400-06-0002, siAR-2 Dharmacon, cat. no. J-003400-07-0002), Opti-MEM and Lipofectamine RNAiMAX (Invitrogen) at the final concentration of 50 nM, and incubated for at least 48 h. Detailed siRNA information is listed in Supplementary Table 4.

### STRING and gene set enrichment analysis

For protein network analysis, the top 51 positive AR regulator hits from the screen were submitted to STRING^37^ (v.11.5). Genes without gene interactions among any of the top 51 hits were removed. Physical subnetwork was chosen. Connections were based on confidence. Line thickness indicates the strength of data support. Genes were clustered by unsupervised Markov Cluster Algorithm (MCL clustering, inflation parameter = 3).

For RNA-seq, gene set enrichment analyses were performed using the preranked method implemented in the fgsea R package (10.18129/B9.bioc.GSEABase), and Hallmark gene sets were downloaded from the molecular signatures database (MSigDB), genes were ranked by the Wald-statistics from DESeq2.

### RNA extraction and qPCR

RNA was extracted from the cells per manufacturer’s protocol using the Zymo Quick-RNA extraction kit (cat. no. R1054). cDNA was prepared using SuperScript III First-Strand Synthesis System (cat. no. 18080). mRNA expression was measured using primers (Supplementary Table 5) and SYBR Green Real-Time PCR Master Mixes (Thermo) in the QuantStudio Flex Real-Time PCR system.

### Western blot, nuclear and cytoplasmic fractionation, and IP

Protein was extracted with either RIPA buffer with protease inhibitor (Thermo, cat. no. 78430). Nuclear and cytoplasmic extracts were prepared using the NE-PER Nuclear and Cytoplasmic Extraction Reagents kit (Thermo Scientific, cat. no. 78833) according to the manufacturer’s instructions. Protein concentrations were quantified by BCA assay before downstream analysis. Western blots were performed as described previously^62^. For immunoprecipitation, protein lysates were incubated with primary antibody overnight and performed as previous described^62^. Antibody information is listed in Supplementary Table 6. Each experiment was performed twice to determine reproducibility, representative images were shown.

### Mass spectrometry

A total of 1 × 10^7^ 22RV1 cells were lysed and then reduced and alkylated at 90 °C for 10 min while shaken at 1,000 rpm per manufacturer’s protocol (PreOmics iST Kit). Clarified protein samples (100 μg) were digested enzymatically using Trypsin/Lys-C mix at 37 °C for 90 min and peptides were desalted and dried down in a speedvac overnight at room temperature (CentriVap, Labconco). Peptide samples were resuspended in Solvent A (2% acetonitrile (ACN), 0.1% formic acid (FA)) and quantified using the Pierce Quantitative Peptide Assay (Thermo Scientific). A total of 1 μg of peptides from each sample were loaded on to an EASY-Spray nanocolumn (Thermo Fisher Scientific, cat. no. ES900) installed on Dionex Ultimate 3000 NanoRSLC coupled with Q-Exactive Plus mass spectrometer (Thermo Fisher Scientific). Peptides were separated over a 95-min gradient of ACN ranging from 2% to 30% ACN followed by a quick ramp up to 80% ACN. Tandem mass spectrometry (MS/MS) scans were performed over mass range of *m*/*z* 350–1,500 with resolution of 17,500. The isolation window was set to 4.0 *m*/*z* and the charge state was set to 2.

The MS/MS raw data (.raw files) were processed using MSFragger within FragPipe^63^ with default label free quantification settings and searched against the human uniprot database. The contaminant and decoy protein sequences were added to the search database using Fragpipe. The computational tools PeptideProphet and ProteinProphet were used for statistical validation of results and subsequent mapping of the peptides to the proteins respectively with 1% false discovery rate. The label free quantification intensities were considered for secondary data analysis. Features having missing values of more than 50% were eliminated, and *k*-nearest neighbor was used to impute the missing values. The data were log transformed, normalized and subjected to Welch’s *t*-tests (Source Data; Fig. 3). Proteins with *P* values less than 0.05 were used to identify the differentially expressed (DE) proteins. Statistical analysis and data visualization were performed in R studio and MetaboAnalyst 5.0^64^.

### IHC and staining score evaluation

The prostate tumor microarray (TMA) with correlated clinical information from 120 clinically localized PCa patients was constructed by the University of Michigan Center for Translational Pathology as previously reported^65^. IHC was performed as described previously^66^. Briefly, paraffin-embedded TMA slides were deparaffinized and rehydrated following standard protocols. Antigen retrieval was carried out with HIER EDTA buffer. Endogenous peroxidase activity was blocked using 1% hydrogen peroxide. Slides were probed overnight at 4 °C with anti-PTGES3 (1:20, Sigma cat. no. HPA038672), washed, incubated with biotinylated secondary anti-rabbit antibody (Jackson cat. no. 111-065-144, 1:2,000), followed by incubation with streptavidin-HRP (Invitrogen cat. no. SA10001, 1:250). Staining was visualized using DAB developing kit (Vector Laboratories) and nuclei were counterstained with hematoxylin (Vector Laboratories). The stained TMA slide was scanned by a Leica Aperio AT2 scanner and digital images were evaluated independently and scored blindly by two pathologists (B.A.S. and J.Z.). IHC scores of nuclear PTGES3 were calculated by IHC signal intensity (0–1 as weak and 2–3 as strong).

### Recombinant protein expression and purification

Protein expression constructs, expression and purification of recombinant mouse AR constructs, including the DNA-binding domain (AR_DBD_, residues 548–651) and a variant lacking the N-terminal domain (AR_ΔNTD_, residues 548–909) were performed as described previously^50,51^. Full-length human PTGES3 was engineered with a Smt3 N-terminal tag and cloned into pET28a (Gene Universal), transformed into BL21DE3 codon plus cells (Novagen) and grown in lysogeny broth, with expression induced by addition of 0.2 mM isopropyl-β-D-thiogalactoside and overnight shaking at 18 °C. Cells were lysed by French press (Constant Systems) and supernatants purified by Ni-NTA (Qiagen), followed by purification by size exclusion chromatography (Superdex 75, Cytiva), overnight cleavage of the Smt3 tag by Ulp1 (when assaying the tagless variant) and final purification by size exclusion chromatography (Superdex 75, Cytiva) in a final buffer of 500 mM NaCl, 20 mM Hepes pH 7.5, 1 mM Tris(2-carboxyethyl) phosphine (TCEP), 5% glycerol and 0.005% IGEPAL CA-630.

### DNA-binding assays

Duplex DNAs (36-base-pair) with 5′ fluorescein-labels were purchased from Integrated DNA Technologies. The sense strand of the duplex DNAs had the following sequences, with AREs in bold: ARE: /56-FAM/TAAAATGTAAACAACGT**AGAACA**TCAGGAACTCCGG, Canonical ARE: /56-FAM/TAAAATGTAAACAACGT**AGAACA**TCA**TGTTCT**CCGG; binding buffer for fluorescence polarization (FP) and electrophoretic mobility shift assays (EMSA) consisted of 150 mM NaCl, 20 mM HEPES pH 7.5, 1 mM TCEP, 1 μM DHT, 10% glycerol and 0.05% IGEPAL CA-630. Unless otherwise indicated, equimolar amounts of AR_ΔNTD_ (FP) or AR_DBD_ (EMSA) and PTGES3 variants were preincubated on ice for 30 min at 8 μM before mixing with the 100 nM of the indicated DNA. For EMSAs, gel shifted products were resolved on 4% to 20% Tris/borate/EDTA (TBE) PAGE, data from *n* = 3 experiments were analyzed by densitometry using Fiji software, and fit to a two-phase specific binding model (GraphPad Prism). To calculate apparent *K*_d_ values for the FP data, a model for receptor depletion was applied, and data presented as mean ± s.d. from *n* = 4 experiments. Data were analyzed using two-way ANOVA, with ****P < 0.0001; NS (not significant), *P* > 0.05.

### Mass photometry

Complex formation between AR_ΔNTD_ and Smt3-PTGES3 in the absence and presence of unlabeled 36-base-pair double-stranded ARE DNA (bearing the sequence TAAAATGTAAACAACGT**AGAACA**TCA**TGTTCT**CCGG, with AREs in bold) was assessed by mass photometry (Refeyn TwoMP). Tagged PTGES3 was used as its molecular weight is within the linear range of the Refeyn TwoMP assay (32 kDa), and because we determined that the Smt3 tag does not interfere with the ability of PTGES3 to alter AR’s DNA binding activity. Individual proteins and AR–PTGES3 complexes were prepared at 5 μM and incubated on ice for 30 min in DNA binding buffer the presence or absence of DNA. Samples were subsequently flash diluted into PBS to a final concentration of 10 nM. Video recordings were performed immediately to detect single particles over the course of 1 min at a rate of 100 frames per second in ratiometric acquisition settings. Ratiometric counts were converted to molecular weight in kilodaltons using a standard curve generated with ovalbumin (43 kDa), conalbumin (75 kDa), aldolase (158 kDa) and thyroglobulin (669 kDa). Data were analyzed with Refeyn DiscoverMP software to produce histograms of mass values representing the bulk population and represented as normalized counts. Data shown are representative of at least three independent measurements.

### Structural modeling of AR–PTGES3 complexes

Structural models of PTGES3 and AR chaperone and DNA-bound complexes were generated using a combination of experimentally derived structural models (PDB: 1XOW; PDB: 1R4I; PDB: 7KRJ)^43,50,67,68^ and Alphafold 3^69^. For Alphafold models, structural models are color coded according to pLDDT confidence estimates, where red is very high and blue is very low confidence. PyMOL (Schrödinger) was used to perform structural alignments and for figure rendering.

### ChIP–qPCR and Dual X ChIP–qPCR

ChIP and Dual X ChIP assays were performed as described previously^62,70^. In brief, 1 × 10^7^ cells were collected, washed and crosslinked with 1% formaldehyde at room temperature for 10 mins and then quenched in 125 mM glycine. For Dual X ChIP crosslinking, cells were treated with 1.5 mM ethylene glycol bis (EGS in PBS, Sigma) for 30 mins, subsequently crosslinked with 1% formaldehyde at room temperature for 10 mins, and quenched with 1.25 M glycine for 5 mins. Genomic DNA extraction and ChIP assay were then performed using Bioruptor Pico sonication (Diagenode) and HighCell# ChIP kit (Diagenode, cat no. C01010061). Antibodies used for ChIP–qPCR are listed in Supplemental Table 6. Bound DNA was quantified by qPCR (SYBR Green master mix, Invitrogen) using the primer sets listed in Supplementary Table 7. The qPCR results are presented as fold enrichment over control IgG antibody and normalized based on the total input (nonprecipitated chromatin). Primers for the GAPDH promoter were used as a negative control.

### RNA sequencing

RNA was extracted from the cells and reverse transcribed to DNA as described above. The QuantSeq 3’mRNA-Seq library prep kit FWD (Lexogen, cat. no. 015.24) was used to prepare NGS gene expression libraries per the manufacturer’s protocol. Quality control was performed by using the Agilent Bioanalyzer 2100 system and the samples were sequenced using an Illumina HiSeq 4000. The entire RNA-seq experiment was done in two biological replicates. The RNA-seq single-end fastq data generated by Illumina HiSeq 4000 sequencing system were first trimmed to remove adapter sequences using Cutadapt v.2.6 with the ‘-q 10 -m 20’ option^71^. After adapter trimming, FASTQC v.0.11.8 was used to evaluate the sequence trimming as well as overall sequence quality. Using the splice-aware aligner STAR^72^ (v.2.7.1a), RNA-seq reads were aligned onto the Human reference genome build GRCh38decoy using the ‘–outSAMtype BAM SortedByCoordinate–outSAMunmapped Within–outSAMmapqUnique 50–sjdbOverhang 65–chimSegmentMin 12–twopassMode Basic’ option and exon–exon junctions, with human gene model annotation from GENCODE v.30. Gene expression quantification of uniquely mapping reads was performed using the ‘featurecount’ function within Rsubread R package^73^ with ‘GTF.featureType = ‘exon,’ GTF.attrType = ‘gene_id,’ useMetaFeatures=TRUE, allowMultiOverlap=FALSE, countMultiMappingReads=FALSE, isLongRead=FALSE, ignoreDup=FALSE, strandSpecific=0, juncCounts=TRUE, genome=NULL, isPairedEnd=FALSE, requireBothEndsMapped=FALSE, checkFragLength=FALSE, countChimericFragments=TRUE, autosort=TRUE’ option. Cross-sample normalization of expression values and differential expression analysis between the PTGES3 knockdown and control was done using DESeq2 R package^74^. Benjamini–Hochberg corrected *P* < 0.05 and log_2_ foldchange >1 or < −1 were considered statistically significant. All pipelines for RNA-seq data processing are described previously^75^ and available at https://github.com/haolongli87/ptges3_resources.

### Chromatin immunoprecipitation sequencing

Cells were fixed with 1% formaldehyde in PBS for 10 min at room temperature and then quenched in 125 mM glycine. Nuclei were sequentially isolated using ice-cold LB1 buffer (50 mM HEPES-KOH, pH 7.5, 140 mM NaCl, 1 mM EDTA, 10% glycerol, 0.5% NP-40, 0.25% Triton X-100), followed by ice-cold LB2 buffer (10 mM Tris-HCl, pH 8.0, 200 mM NaCl, 1 mM EDTA, 0.5 mM EGTA). Subsequently, nuclear lysis was performed on ice for 10 min using ChIP lysis buffer (1% SDS, 5 mM EDTA, 50 mM Tris-HCl, pH 8.1). Chromatin was sheared to approximately 300-bp fragments with a Bioruptor Plus Sonicator (Diagenode). Immunoprecipitation was carried out using an anti-AR antibody (ab108341). Protein–DNA complexes were reverse crosslinked overnight at 65 °C. ChIP and input DNA samples were purified the following day with the QIAquick PCR Purification Kit (Qiagen, cat. no. 28104). Sequencing libraries were constructed from approximately 0.8 ng ChIP DNA using the ThruPLEX DNA-Seq Prep Kit (Takara Bio, cat. no. R400675) with 12 cycles of PCR amplification. Libraries underwent NGS (100 bp, paired-end) on an Illumina NovaSeq X instrument at the Fred Hutch Genomics Core.

### ChIP–seq data analyses

Raw FASTQ sequencing data were trimmed using Trimmomatic (v.0.39) and aligned to the human genome (hg38) using Burrows–Wheeler Aligner^76^ (BWA-mem, v.0.7.17). Alignments with MAPQ scores lower than 30 were filtered out using SAMtools^77^, and ENCODE blacklisted regions^78^ were excluded using BEDTools^79^ (v.2.31.0). Duplicate reads were identified and removed using Picard MarkDuplicates (v.2.25.1) (http://broadinstitute.github.io/picard). Peak calling was performed using MACS3^80^ (v.3.0.0) with a *Q* value cutoff of 0.01. Normalized BigWig files were generated by bamCoverage from deepTools^81^ (v.3.5.4) using RPGC normalization. Coverage heatmaps centered around genomic features of interest were produced using the computeMatrix and plotHeatmap modules within deepTools (v.3.5.4). Visualization snapshots were obtained using Integrative Genomics Viewer^82^ (IGV, v.2.19.1). Motif analyses utilized HOMER^83^ (v.4.11), applying the Known Motif Discovery approach.

### ATAC-seq

ATAC-seq was performed as described previously^84,85^ with the following modifications. Cells were resuspended in buffer (Illumina, cat. no. 20034198), incubated on ice for 10 min and lysed using a dounce homogenizer. A total of 50,000 nuclei were incubated with 25 µl 2× TD Buffer and 1.25 µl Transposase (Illumina Tagment Enzyme/Buffer, cat. no. 20034210) shaking at 300 rpm at 37 °C for 30 min. Zymo DNA Clean and Concentrator 5 kit (cat. no. D4014) was then used to purify DNA. Transposed DNA was amplified using PCR master mix and indexes from Nextera DNA Library Prep kit (cat. no. 15028211) for five cycles and then assessed using qPCR. Final cleanup was performed using 1.8× AMPure XP beads (cat. no. A63881) and libraries quantified using the DNA High Sensitivity Agilent 2100 Bioanalyzer System. Samples were sequenced at the UCSF Core Facility on a HiSeq4000 as PE100 libraries. The entire experimental setup was performed in two technical replicates.

### ATAC-seq data processing

The ATAC-seq paired-end fastq files were first trimmed to remove Illumina Nextera adapter sequence using Cutadapt v2.6^86^ with the ‘-q 10 -m 20’ option. FASTQC v.0.11.8^87^ was used to evaluate sequence trimming and overall sequence quality. Bowtie2 v.2.3.5.1^88^ was used to align ATAC-seq reads against the Human reference genome build GRCh38decoy using the ‘–very-sensitive’ option. Uniquely mapped reads were obtained in SAM format. Samtools version 1.9^89^ was used to convert SAM to BAM file and sort the BAM file. Picard (https://broadinstitute.github.io/picard/) was then used to flag duplicate reads using the MarkDuplicates tool using ‘REMOVE_DUPLICATES=true’ option. The resulting BAM file reads position were then corrected by a constant offset to the read start (‘+’ stranded +4 bp, ‘−’ stranded −5 base pairs) using deepTools2 v.3.3.2^81^ with the ‘alignmentSieve–ATACshift’ option. This resulted in the final aligned, de-duplicated BAM file that was used in all downstream analyses. ATAC-seq peak calling was performed using MACS2 v.2.2.5^80^ to obtain narrow peaks with the ‘callpeak -f BAMPE -g hs –qvalue 0.05–nomodel -B–keep-dup all–call-summits’ option. The resulting peaks that map to the mitochondrial genome or genomic regions listed in the ENCODE hg38 blacklist (https://www.encodeproject.org/annotations/ENCSR636HFF/) or peaks that extend beyond the ends of chromosomes were filtered out. Nonoverlapping unique ATAC-seq narrow peak regions were obtained from all samples analyzed. Only those nonoverlapping unique peak regions present in at least two samples were considered for further analysis. Sequencing reads mapped to these nonoverlapping unique regions were counted using ‘featurecount’ function within Rsubread^72^ R package with the ‘isPairedEnd=TRUE, countMultiMappingReads=FALSE, maxFragLength=100, autosort=TRUE’ option. Further library-size normalization of the feature counts and differential OCRs between sgPTGES3 and sgCTRL were obtained using the DESeq2^74^ R package. Only those peak regions with Benjamini–Hochberg corrected *P* < 0.05 and log_2_ foldchange >1 or <−1 were considered statistically significant. The ATAC-seq peaks were annotated using ChIPseeker^90^ R package based on hg38 GENCODE v.30 annotations.

#### Over-representation analysis

The genes nearest to the ATAC-seq peaks that mapped within the promoter regions of the corresponding gene were considered for this analysis. These genes were tested for enrichment against Hallmark gene sets in Molecular Signature Database (MSigDB) v.7.0. A hypergeometric test-based over-representation analysis was used for this purpose. A cutoff threshold of false discovery rate ≤ 0.01 was used to obtain the significantly enriched gene sets.

##### TF binding analysis

The differentially accessible ATAC-seq peaks that mapped to promoter and intergenic regions were used for TF binding analysis. The MEME tool^91^ was used to discover TF binding motifs de novo. These potential TF binding motifs were annotated for known TF motif from the JASPAR^92^ database using the TomTom tool within the MEME tool suite. All pipelines for ATAC data processing are described previously^93^ and are available at https://github.com/haolongli87/ptges3_resources.

### Clinical cohort analysis

Publicly available gene expression data from a matched cohort established previously^59^ or mCRPC was downloaded from cBioportal (20 May 2021)^94–96^. Samples were grouped based on expression levels above or below the 25th percentile for PTGES3 separately for poly-A or capture-based RNA-seq, and the capture-based results were used if a sample had sequencing data available from both methods. Differences in survival between groups were visualized using the Kaplan–Meier method and a log-rank test was used to test for differences in survival, using the survival and survminer R packages^97,98^. Hazard ratios were calculated using Cox proportional hazards regression.

### Tumor models

All animal studies were conducted in compliance with UCSF Institutional Animal Care and Use Committee (IACUC) guidelines. NOD-SCID-Gamma (NSG) mice (Jackson Laboratory, strain code 005557) were housed in a pathogen-free barrier facility under a 12 h light/12 h dark cycle at 18–23 °C and 40–60% humidity. All in vivo experiments used male mice aged 6–8 weeks. Doxycycline-inducible LNCaPi cells established previously^99^ were lentiviral transfected with constructs encoding sgPTGES3 or sgCTRL and puromycin selected. Cells were mixed with Matrigel (Corning, cat. no. 354230, 1:1) and 2 × 10^6^ cells were injected subcutaneously on each flank of male NSG mice. Once the tumors were palpable, mice were randomized into two groups receiving either doxycycline diet (Bio-Serv, cat. no. S3888) or control diet. Tumors were measured using digital calipers. Tumor volume was calculated using the equation: volume = length × width^2^ × 0.52, where the length represents the longer axis. Average tumor volume was plotted and two-way ANOVA was used to measure statistical significance denoted by asterisk (*P < 0.05, **P < 0.01, ***P < 0.001). The mice were humanely euthanized per UCSF LARC protocol once the tumor volume reached 1,000 mm^3^.

### WST-1, clonogenic and IncuCyte assays

Cell viability assays were performed as described previously^62^ using the cell proliferation reagent WST-1 (Sigma) according to the manufacturerʼs protocol. Clonogenic assays were performed as described previously^99^. In brief, 1,000 cells were seeded in a six-well plate and treated as indicated for 10 days. The cell colonies were then washed with PBS and fixed/stained with 25% methanol plus crystal violet (0.05% w/v). Images were scanned and analyzed using a GelCount (Oxford Optronix). Average number of colonies was counted. For IncuCyte experiment, cells labeled with Nuclight red were seeded to 96-well plates. The time relapse images were captured and analyzed with by the Incucyte S3 live-cell analysis system.

### Proximity ligation assays

Proximity ligation assays were performed using the Duolink in situ red starter kit mouse/rabbit (Sigma). LNCaP cells were fixed with 4% PFA and permeabilized by 0.2% Triton X-100. Fixed cells were incubated with primary antibodies overnight at 4 °C. Antibodies used for proximity ligation assays are listed in Supplementary Table 6. Secondary probe, ligation and amplification reactions were performed following the manufacturer’s instructions. Fluorescence images were captured by Zeiss fluorescent microscope (Carl Zeiss).

### Statistical analysis and reproducibility

Spearman’s correlation was used to determine statistical significance for all the correlation plots. For gene expression and correlation, the Wilcoxon rank-sum test was used to test for differences between two groups, unless otherwise stated. Unpaired *t* test were used to determine statistical analysis for the column plots, denoted by asterisk (*P < 0.05, **P < 0.01, ***P < 0.001). Two-way ANOVA was used to determine statistical significance in the in vivo data. In RNA-seq data, the Benjamini–Hochberg test was performed. Corrected *P* < 0.05 and log_2_ foldchange >0.5 or <0.5 were considered statistically significant. In ATAC-seq data peak regions with Benjamini–Hochberg corrected *P* < 0.05 and log_2_ foldchange >0.5 or <0.5 were considered statistically significant. No statistical methods were used to predetermine sample sizes for any experiments. For all analyses, data distribution was assumed to be normal, but this was not formally tested.

### Reporting summary

Further information on research design is available in the Nature Portfolio Reporting Summary linked to this article.

## Online content

Any methods, additional references, Nature Portfolio reporting summaries, source data, extended data, supplementary information, acknowledgements, peer review information; details of author contributions and competing interests; and statements of data and code availability are available at 10.1038/s41588-025-02388-8.

## Supplementary information

Reporting Summary

Supplementary Tables 1–7 Table 1: CRISPR knock-in oligos. Table 2: cloning primers. Table 3: validation sgRNA primers. Table 4: siRNA. Table 5: real-time PCR primers. Table 6: antibodies. Table 7: ChIP–qPCR primers.

## Source data

Source Data Fig. 2 Statistical source data from applicable figure panels in Fig. 2.

Source Data Fig. 3 Statistical source data from applicable figure panels in Fig. 3.

Source Data Fig. 4 Statistical source data from applicable figure panels in Fig. 4.

Source Data Fig. 6 Statistical source data from applicable figure panels in Fig. 6.

Source Data unprocessed western blots for Figs. 1–4 Unprocessed western blots.

Source Data unprocessed western blots for Extended Data figures Unprocessed western blots.

## Data Availability

All data supporting this study are included in the manuscript and Supplementary Information. Raw NGS data are deposited in the Gene Expression Omnibus (GEO): sgPTGES3 ATAC-seq (GSE180034), sgPTGES3 mRNA-seq (GSE180035), sgPTGES3 ChIP–seq (GSE292612). CRISPR screen data are available in the Sequence Read Archive (SRA) under accession PRJNA1284972. Mass spectrometry proteomics data are deposited in PRIDE (PXD066297). Public ChIP–seq data were obtained from GEO (GSE120741). Source data are provided with this paper.

## Code availability

All code for data processing and figure generation is available via GitHub at https://github.com/haolongli87/ptges3_resources and via Zenodo at 10.5281/zenodo.15678437 (ref. ^100^).
