# Supplementary material for: Genome-scale CRISPR screens identify PTGES3 as a direct modulator of androgen receptor function in advanced prostate cancer
Source: Nat Genet. 2025 Nov 5;57(12):3027–38. doi: 10.1038/s41588-025-02388-8 (PMC12695660; doi:10.1038/s41588-025-02388-8)
Supplement: Supplementary file 1 — Reporting Summary [file 41588_2025_2388_MOESM1_ESM.pdf]

nature portfolio

Corresponding author(s): Haolong Li and Luke Gilbert

Last updated by author(s): Aug 16, 2025

Reporting Summary

Nature Portfolio wishes to improve the reproducibility of the work that we publish. This form provides structure for consistency and transparency in reporting. For further information on Nature Portfolio policies, see our Editorial Policies and the Editorial Policy Checklist.

Statistics

For all statistical analyses, confirm that the following items are present in the figure legend, table legend, main text, or Methods section.

n/a	Confirmed	
☐	☒	The exact sample size (n) for each experimental group/condition, given as a discrete number and unit of measurement
☐	☒	A statement on whether measurements were taken from distinct samples or whether the same sample was measured repeatedly
☐	☒	The statistical test(s) used AND whether they are one- or two-sided<br>*Only common tests should be described solely by name; describe more complex techniques in the Methods section.*
☐	☒	A description of all covariates tested
☒	☐	A description of any assumptions or corrections, such as tests of normality and adjustment for multiple comparisons
☐	☒	A full description of the statistical parameters including central tendency (e.g. means) or other basic estimates (e.g. regression coefficient) AND variation (e.g. standard deviation) or associated estimates of uncertainty (e.g. confidence intervals)
☐	☒	For null hypothesis testing, the test statistic (e.g. F, t, r) with confidence intervals, effect sizes, degrees of freedom and P value noted<br>*Give P values as exact values whenever suitable.*
☒	☐	For Bayesian analysis, information on the choice of priors and Markov chain Monte Carlo settings
☒	☐	For hierarchical and complex designs, identification of the appropriate level for tests and full reporting of outcomes
☐	☒	Estimates of effect sizes (e.g. Cohen's d, Pearson's r), indicating how they were calculated

Our web collection on statistics for biologists contains articles on many of the points above.

Software and code

Policy information about availability of computer code

Data collection	Not applicable
Data analysis	A description of all custom code used for analysis is provided in the methods.

For manuscripts utilizing custom algorithms or software that are central to the research but not yet described in published literature, software must be made available to editors and reviewers. We strongly encourage code deposition in a community repository (e.g. GitHub). See the Nature Portfolio guidelines for submitting code & software for further information.

Data

Policy information about availability of data

All manuscripts must include a data availability statement. This statement should provide the following information, where applicable:

- Accession codes, unique identifiers, or web links for publicly available datasets
- A description of any restrictions on data availability
- For clinical datasets or third party data, please ensure that the statement adheres to our policy

Data Availability: All data generated or analyzed during this study are included article and supplementary information or have been publicly deposited as indicated.

Research involving human participants, their data, or biological material

Policy information about studies with human participants or human data. See also policy information about sex, gender (identity/presentation), and sexual orientation and race, ethnicity and racism.

Reporting on sex and gender	*Use the terms sex (biological attribute) and gender (shaped by social and cultural circumstances) carefully in order to avoid confusing both terms. Indicate if findings apply to only one sex or gender; describe whether sex and gender were considered in study design; whether sex and/or gender was determined based on self-reporting or assigned and methods used. Provide in the source data disaggregated sex and gender data, where this information has been collected, and if consent has been obtained for sharing of individual-level data; provide overall numbers in this Reporting Summary. Please state if this information has not been collected. Report sex- and gender-based analyses where performed, justify reasons for lack of sex- and gender-based analysis.*
Reporting on race, ethnicity, or other socially relevant groupings	*Please specify the socially constructed or socially relevant categorization variable(s) used in your manuscript and explain why they were used. Please note that such variables should not be used as proxies for other socially constructed/relevant variables (for example, race or ethnicity should not be used as a proxy for socioeconomic status). Provide clear definitions of the relevant terms used, how they were provided (by the participants/respondents, the researchers, or third parties), and the method(s) used to classify people into the different categories (e.g. self-report, census or administrative data, social media data, etc.) Please provide details about how you controlled for confounding variables in your analyses.*
Population characteristics	*Describe the covariate-relevant population characteristics of the human research participants (e.g. age, genotypic information, past and current diagnosis and treatment categories). If you filled out the behavioural & social sciences study design questions and have nothing to add here, write "See above."*
Recruitment	*Describe how participants were recruited. Outline any potential self-selection bias or other biases that may be present and how these are likely to impact results.*
Ethics oversight	*Identify the organization(s) that approved the study protocol.*

Note that full information on the approval of the study protocol must also be provided in the manuscript.

Field-specific reporting

Please select the one below that is the best fit for your research. If you are not sure, read the appropriate sections before making your selection.

☒ Life sciences ☐ Behavioural & social sciences ☐ Ecological, evolutionary & environmental sciences

For a reference copy of the document with all sections, see nature.com/documents/nr-reporting-summary-flat.pdf

Life sciences study design

All studies must disclose on these points even when the disclosure is negative.

Sample size	No sample size calculations were performed. All samples sizes were chosen according to the accepted standard in the field. Data displayed is 2-3 independent biological experiments with 2-3 technical replicates per biological experiment except as noted. For clinical sample analysis we used all available samples.
Data exclusions	No data related to experiments in the manuscript were excluded.
Replication	We used 2-3 biological replicates per experiment generally with as 2-3 technical replicates per experiment. Exact number of samples was previously provided to the Nature Communications and senior editor Dr. Sundaram. All attempts at replication were successful.
Randomization	Randomization is not relevant to the clinical or experimental studies that were performed. Randomization is not standardly performed in this field of biology research.
Blinding	Blinding was not performed however a number of experiments were independently replicated by co-authors to ensure reproducibility. Blinded experiments are not standardly performed in this field of experimental biology research.

Reporting for specific materials, systems and methods

We require information from authors about some types of materials, experimental systems and methods used in many studies. Here, indicate whether each material, system or method listed is relevant to your study. If you are not sure if a list item applies to your research, read the appropriate section before selecting a response.

Materials & experimental systems

n/a	Involved in the study	
☐	☒	Antibodies
☐	☒	Eukaryotic cell lines
☒	☐	Palaeontology and archaeology
☐	☒	Animals and other organisms
☒	☐	Clinical data
☒	☐	Dual use research of concern
☒	☐	Plants

Methods

n/a	Involved in the study	
☐	☒	ChIP-seq
☐	☒	Flow cytometry
☒	☐	MRI-based neuroimaging

Antibodies

Antibodies used	All antibodies information were reported in Supplementary table 3.
Validation	Antibody specificity for primary antibodies was validated by CRISPRi knockdown experiments. All antibodies were raised for human proteins and used to detect human proteins.

Eukaryotic cell lines

Policy information about cell lines and Sex and Gender in Research

Cell line source(s)	293T, LNCaP, C42B, VCaP, 22Rv1, PC3, DU145, UMUC3, T24, OVCAR8, A549, K562, and Jurkat cell lines were purchased from ATCC. MR49F cells were a gift from the laboratory of A. Zoubeidi (Vancouver Prostate Center).
Authentication	Cell lines were authenticated by STR analysis yearly.
Mycoplasma contamination	All cell lines tested negative for mycoplasma. Mycoplasma testing was performed every 3-6 months.
Commonly misidentified lines (See ICLAC register)	No commonly misidentified cell lines were used in this study.

Animals and other research organisms

Policy information about studies involving animals; ARRIVE guidelines recommended for reporting animal research, and Sex and Gender in Research

Laboratory animals	NSG male mice 6-8 weeks of age were used for all experiments.
Wild animals	No wild animals were used in this study.
Reporting on sex	To establish prostate cancer xenograft model, male NSG mice were used.
Field-collected samples	No field-collected samples were used in this study.
Ethics oversight	UCSF IACUC approval was obtained prior to all experiments.

Note that full information on the approval of the study protocol must also be provided in the manuscript.

Plants

Seed stocks	*Report on the source of all seed stocks or other plant material used. If applicable, state the seed stock centre and catalogue number. If plant specimens were collected from the field, describe the collection location, date and sampling procedures.*
Novel plant genotypes	*Describe the methods by which all novel plant genotypes were produced. This includes those generated by transgenic approaches, gene editing, chemical/radiation-based mutagenesis and hybridization. For transgenic lines, describe the transformation method, the number of independent lines analyzed and the generation upon which experiments were performed. For gene-edited lines, describe the editor used, the endogenous sequence targeted for editing, the targeting guide RNA sequence (if applicable) and how the editor was applied.*
Authentication	*Describe any authentication procedures for each seed stock used or novel genotype generated. Describe any experiments used to assess the effect of a mutation and, where applicable, how potential secondary effects (e.g. second site T-DNA insertions, mosiacism, off-target gene editing) were examined.*

ChIP-seq

Data deposition

☒ Confirm that both raw and final processed data have been deposited in a public database such as GEO.

☒ Confirm that you have deposited or provided access to graph files (e.g. BED files) for the called peaks.

Data access links<br>*May remain private before publication.*	sgPTGES3 ChIP-seq<br>• GEO accession: GSE292612<br>• Link: https://www.ncbi.nlm.nih.gov/geo/query/acc.cgi?acc=GSE292612
Files in database submission	GSM8862405 LNCaP_sgCtrl, biological replicate 1, technical replicate 1 (ChIP-AR)<br>GSM8862406 LNCaP_sgCtrl, biological replicate 1, technical replicate 2 (ChIP-AR)<br>GSM8862407 LNCaP_sgCtrl, biological replicate 2, technical replicate 1 (ChIP-AR)<br>GSM8862408 LNCaP_sgCtrl, biological replicate 2, technical replicate 2 (ChIP-AR)<br>GSM8862409 LNCaP_sgPTGES3, biological replicate 1, technical replicate 1 (ChIP-AR)<br>GSM8862410 LNCaP_sgPTGES3, biological replicate 1, technical replicate 2 (ChIP-AR)<br>GSM8862411 LNCaP_sgPTGES3, biological replicate 2, technical replicate 1 (ChIP-AR)<br>GSM8862412 LNCaP_sgPTGES3, biological replicate 2, technical replicate 2 (ChIP-AR)<br>GSM8862413 LNCaP_sgCtrl, biological replicate 1 (input)<br>GSM8862414 LNCaP_sgCtrl, biological replicate 2 (input)<br>GSM8862415 LNCaP_sgPTGES3, biological replicate 1 (input)<br>GSM8862416 LNCaP_sgPTGES3, biological replicate 2 (input)
Genome browser session<br>(e.g. UCSC)	*Provide a link to an anonymized genome browser session for "Initial submission" and "Revised version" documents only, to enable peer review. Write "no longer applicable" for "Final submission" documents.*

Methodology

Replicates	The ChIP-seq experiments were performed with two biological replicates per condition, each containing two technical replicates, for a total of four individual experiments.
Sequencing depth	Per sample per replicate 49-65M reads
Antibodies	anti-AR antibody (ab108341)
Peak calling parameters	Peak calling was performed using MACS3 (v3.0.0)77 with a q-value cutoff of 0.01.
Data quality	Raw FASTQ sequencing data were trimmed using Trimmomatic (v0.39) and aligned to the human genome (hg38) using Burrows-Wheeler Aligner (BWA-mem, v0.7.17). Alignments with MAPQ scores lower than 30 were filtered out using SAMtools, and ENCODE blacklisted regions were excluded using BEDTools (v2.31.0). Duplicate reads were identified and removed using Picard MarkDuplicates (v2.25.1) (http://broadinstitute.github.io/picard)
Software	Raw FASTQ sequencing data were trimmed using Trimmomatic (v0.39) and aligned to the human genome (hg38) using Burrows-Wheeler Aligner (BWA-mem, v0.7.17). Alignments with MAPQ scores lower than 30 were filtered out using SAMtools, and ENCODE blacklisted regions were excluded using BEDTools (v2.31.0). Duplicate reads were identified and removed using Picard MarkDuplicates (v2.25.1) (http://broadinstitute.github.io/picard). Peak calling was performed using MACS3 (v3.0.0) with a q-value cutoff of 0.01. Normalized BigWig files were generated via bamCoverage from deepTools (v3.5.4) using RPGC normalization. Coverage heatmaps centered around genomic features of interest were produced using the computeMatrix and plotHeatmap modules within deepTools (v3.5.4). Visualization snapshots were obtained using Integrative Genomics Viewer (IGV, v2.19.1). Motif analyses utilized HOMER (v4.11), applying the Known Motif Discovery approach.

Flow Cytometry

Plots

Confirm that:

☒ The axis labels state the marker and fluorochrome used (e.g. CD4-FITC).

☒ The axis scales are clearly visible. Include numbers along axes only for bottom left plot of group (a 'group' is an analysis of identical markers).

☒ All plots are contour plots with outliers or pseudocolor plots.

☒ A numerical value for number of cells or percentage (with statistics) is provided.

Methodology

Sample preparation	All flow cytometry data is derived from cell lines purchased from ATCC as listed in the methods. The cells were lifted by trypsin and quenched with growth media. The cells were washed, resuspended in flow buffer, sorted, and analyzed with the Invitrogen Attune NxT flow cytometer or BD FACSAria™ Fusion cell sorter. For genome wide flow based CRISPRi screen, after

	collection, cells were fixed using 3% PFA, quenched by 30 mM glycine/PBS (pH 7.5), and collected for sorting with the BD FACSAria™ Fusion cell sorter.
Instrument	Invitrogen Attune NxT flow cytometer; BD FACSAria™ Fusion cell sorter
Software	Invitrogen Attune NxT flow cytometry software v3.1; BD FACSDiva™ Software; BD flowjo V10
Cell population abundance	A minimum of 10,000 cells were analyzed for all flow cytometry software
Gating strategy	All samples were gated on FSC/SSC to identify and analyze live cells and exclude dead/dying cells or debris. BFP, GFP, or mCherry gates were set using negative and single positive control cells.

☒ Tick this box to confirm that a figure exemplifying the gating strategy is provided in the Supplementary Information.
