# Supplementary figures and images for: Genome-scale CRISPR screens identify PTGES3 as a direct modulator of androgen receptor function in advanced prostate cancer
Source: Nat Genet. 2025 Nov 5;57(12):3027–38. doi: 10.1038/s41588-025-02388-8 (PMC12695660; doi:10.1038/s41588-025-02388-8)

Fig.1b

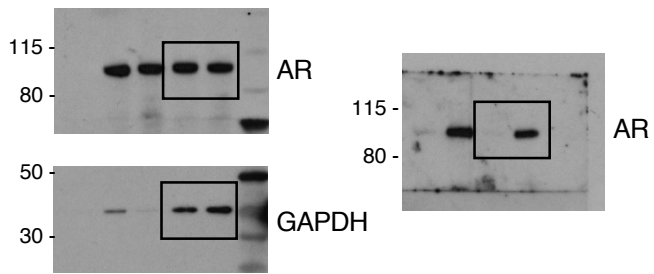

Fig.1f

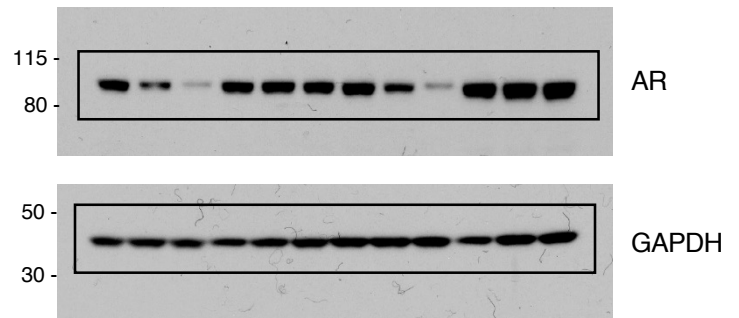

Fig.2e

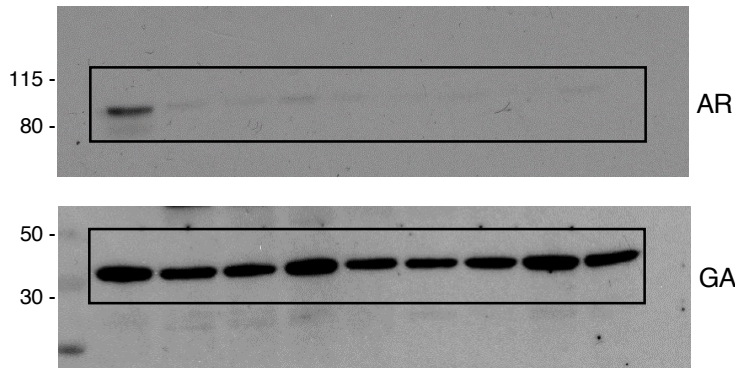

Fig.2f

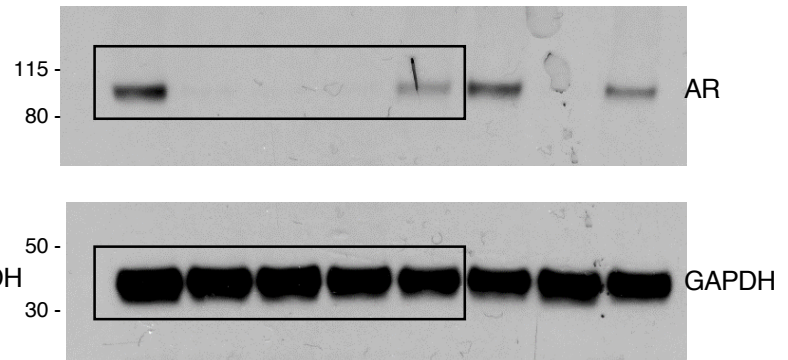

Fig.3b

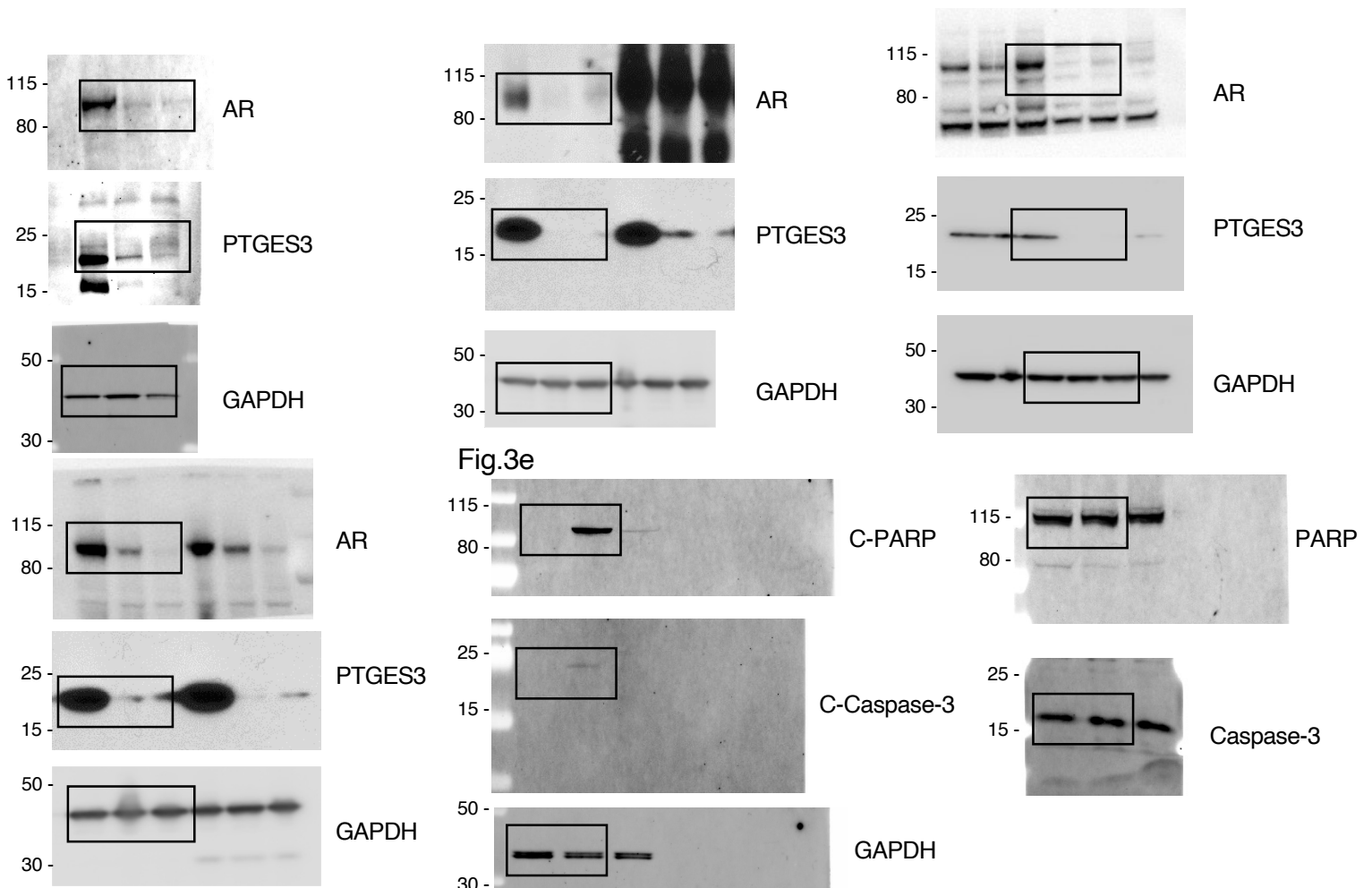

Fig.3g

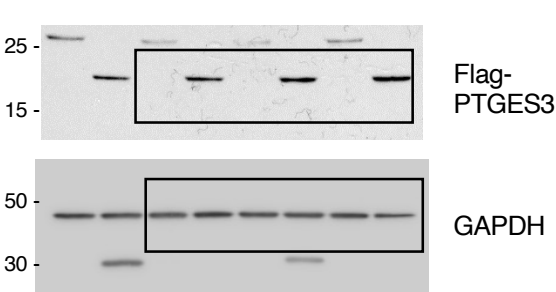

Fig.3h

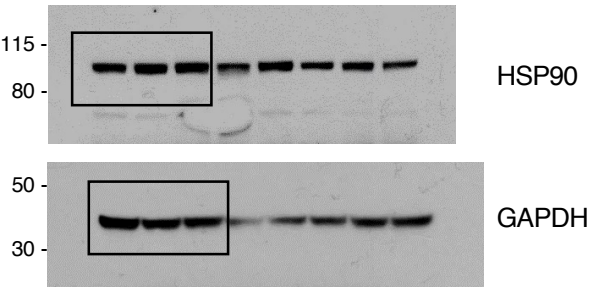

Fig.3h

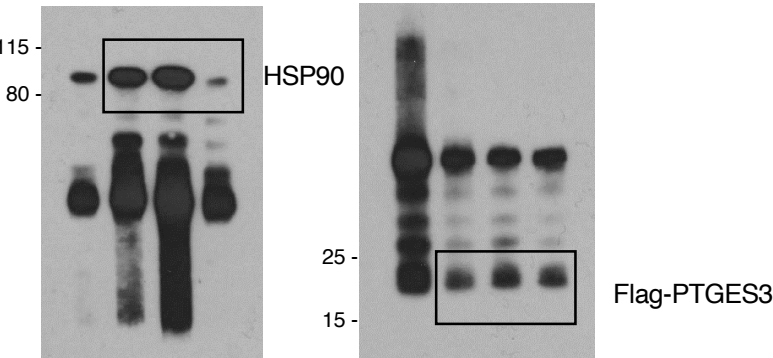

Fig.3i

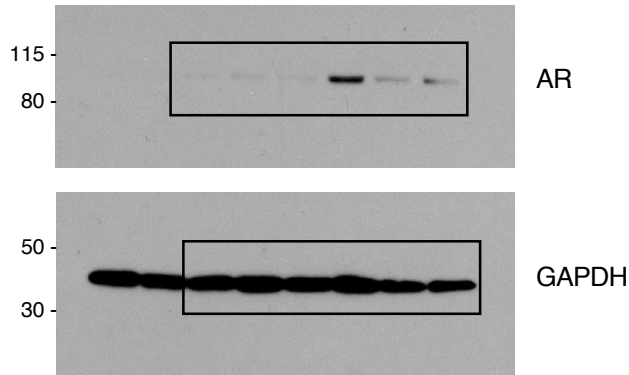

Fig.4b

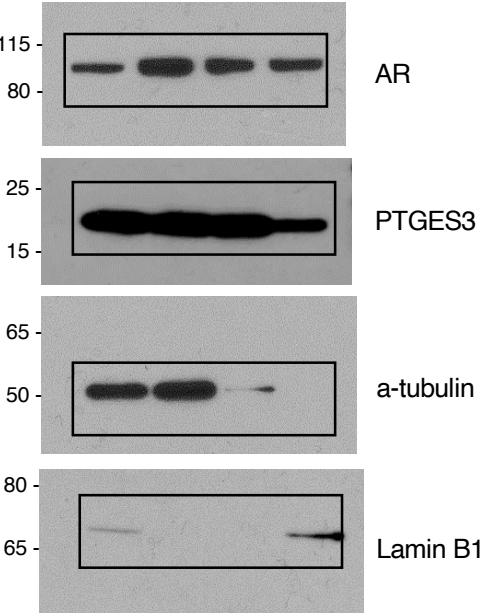

Fig.4c

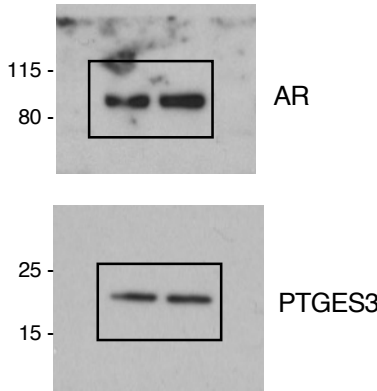

Fig.4e

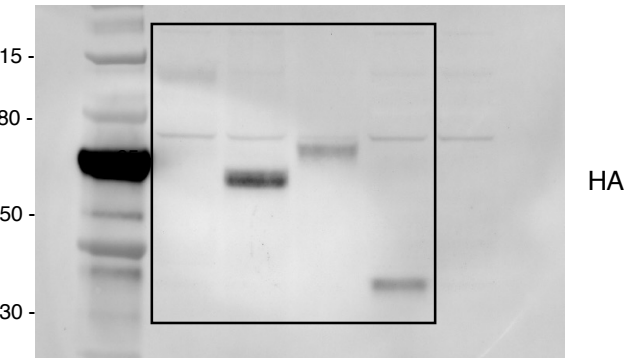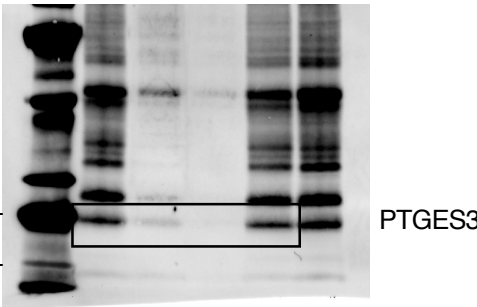

Supplement: Supplementary file 7 — Unprocessed western blots. [file 41588_2025_2388_MOESM7_ESM.pdf]
