# Supplementary material for: Genome-scale CRISPR screens identify PTGES3 as a direct modulator of androgen receptor function in advanced prostate cancer
Source: Nat Genet. 2025 Nov 5;57(12):3027–38. doi: 10.1038/s41588-025-02388-8 (PMC12695660; doi:10.1038/s41588-025-02388-8)

ED Fig.3c

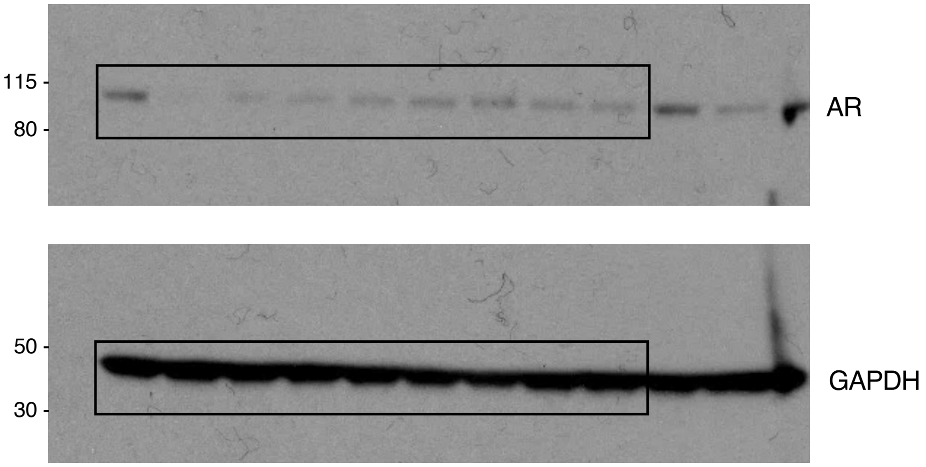

ED Fig.3d

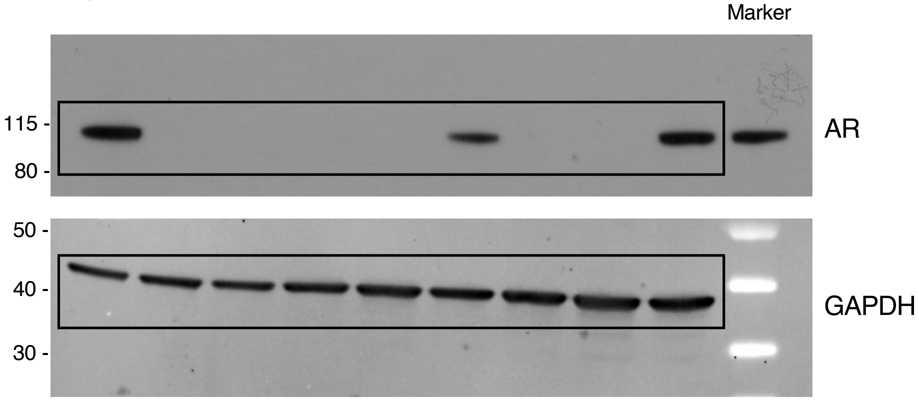

ED Fig.4F

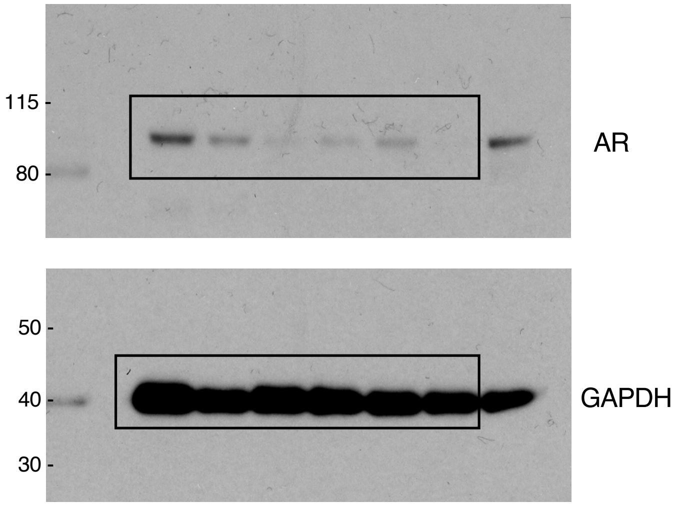

ED Fig.4F

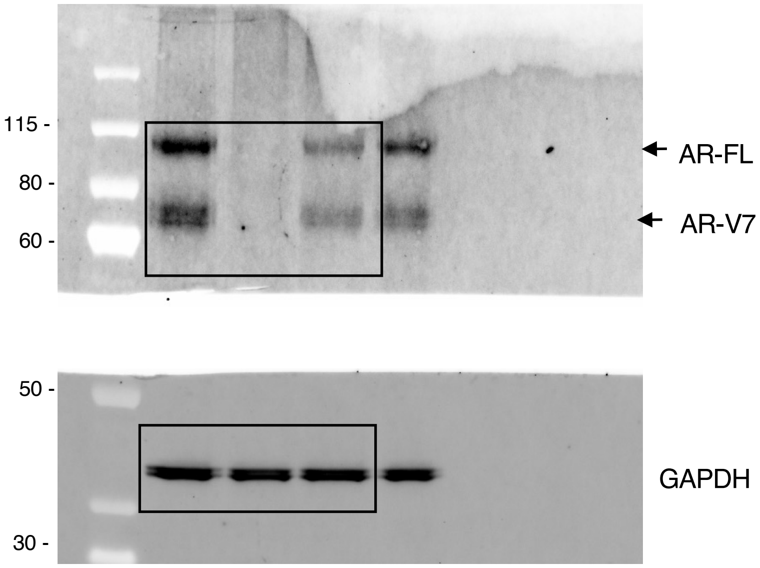

ED Fig.5a

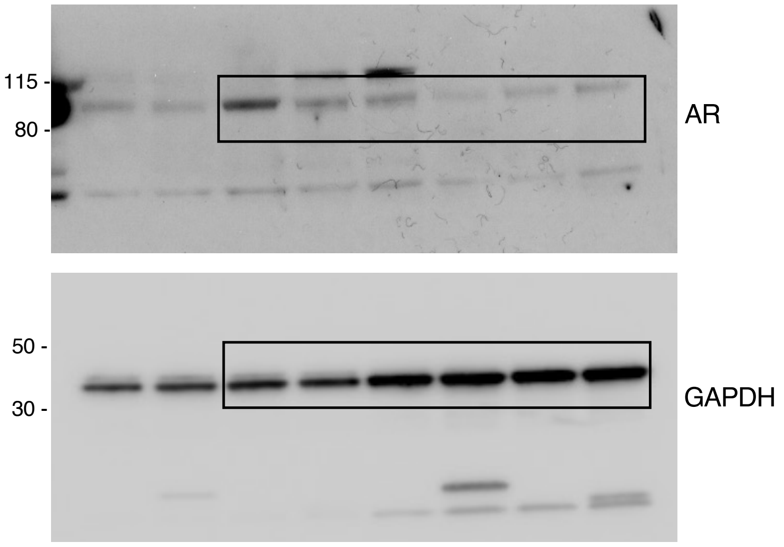

ED Fig.5c

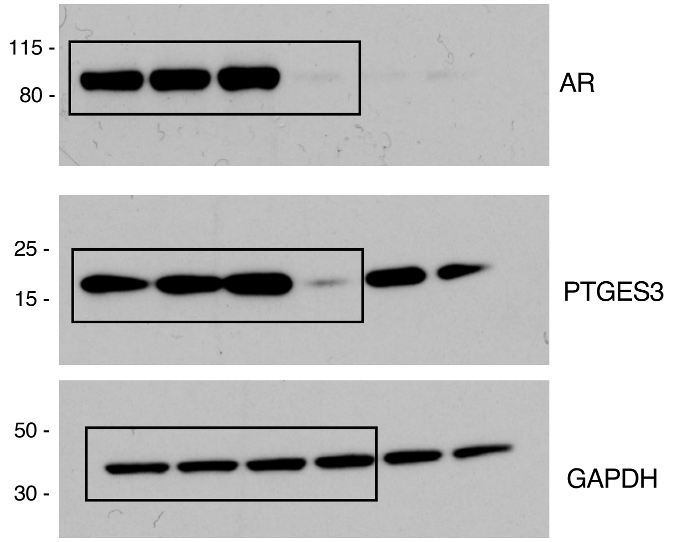

ED Fig.5d

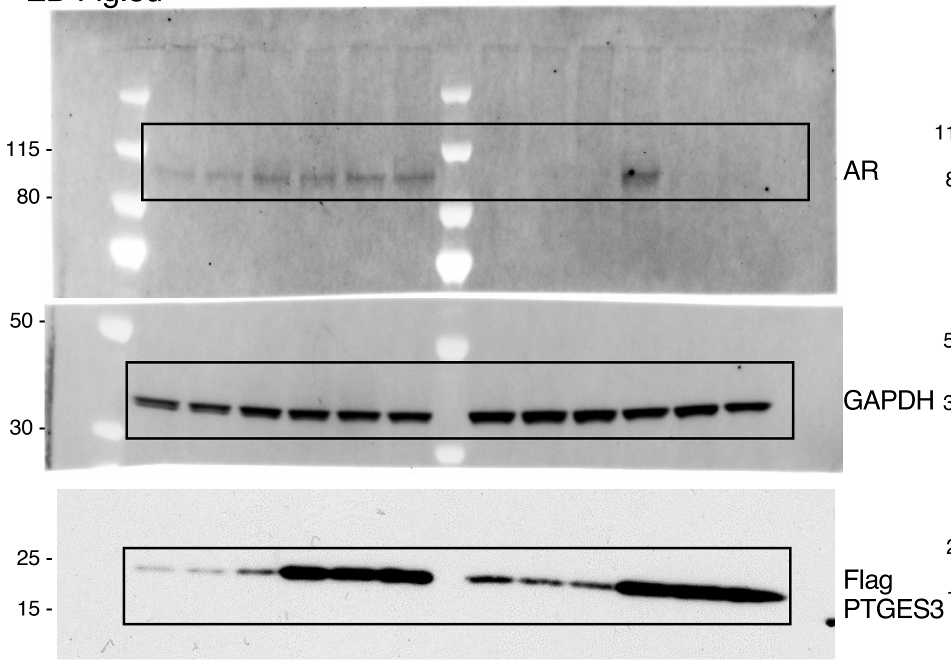

ED Fig.5e

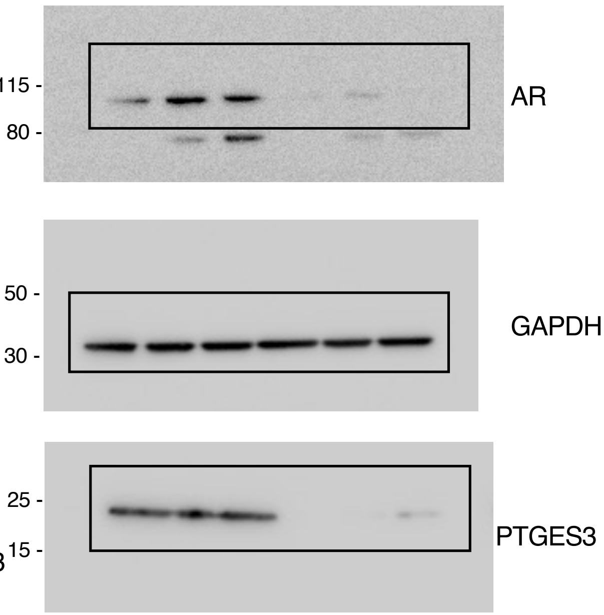

ED Fig.5f

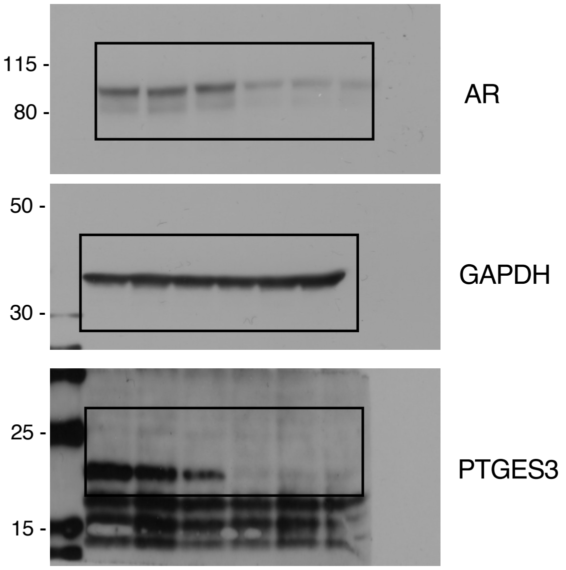

ED Fig.5g

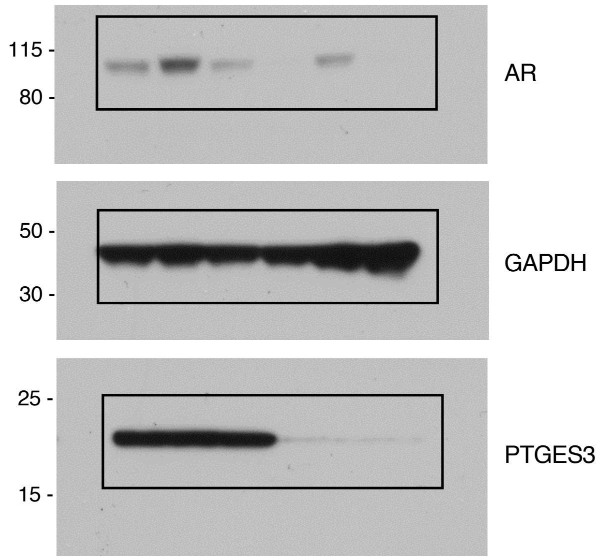

ED Fig. 7a, b

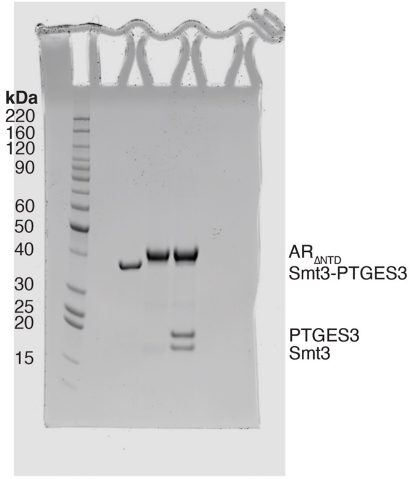

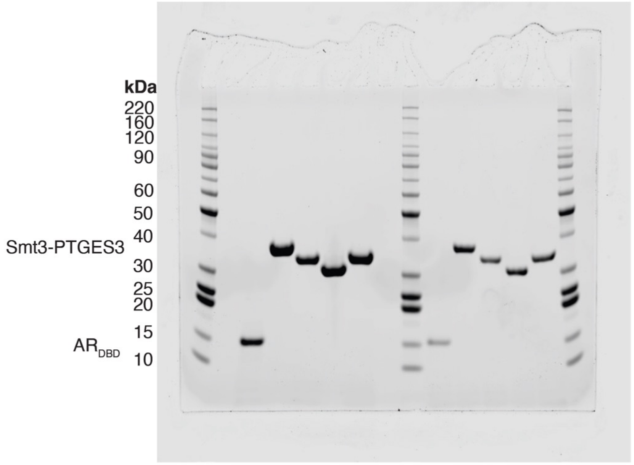

ED Fig. 7g,h

Lanes: 1: 4000; 2: 2000; 3: 1000; 4: 500; 5: 250; 6: 128; 7: 64; 8: 32; 9: 16; 10: 8; 11: 4; 12: 0 nM

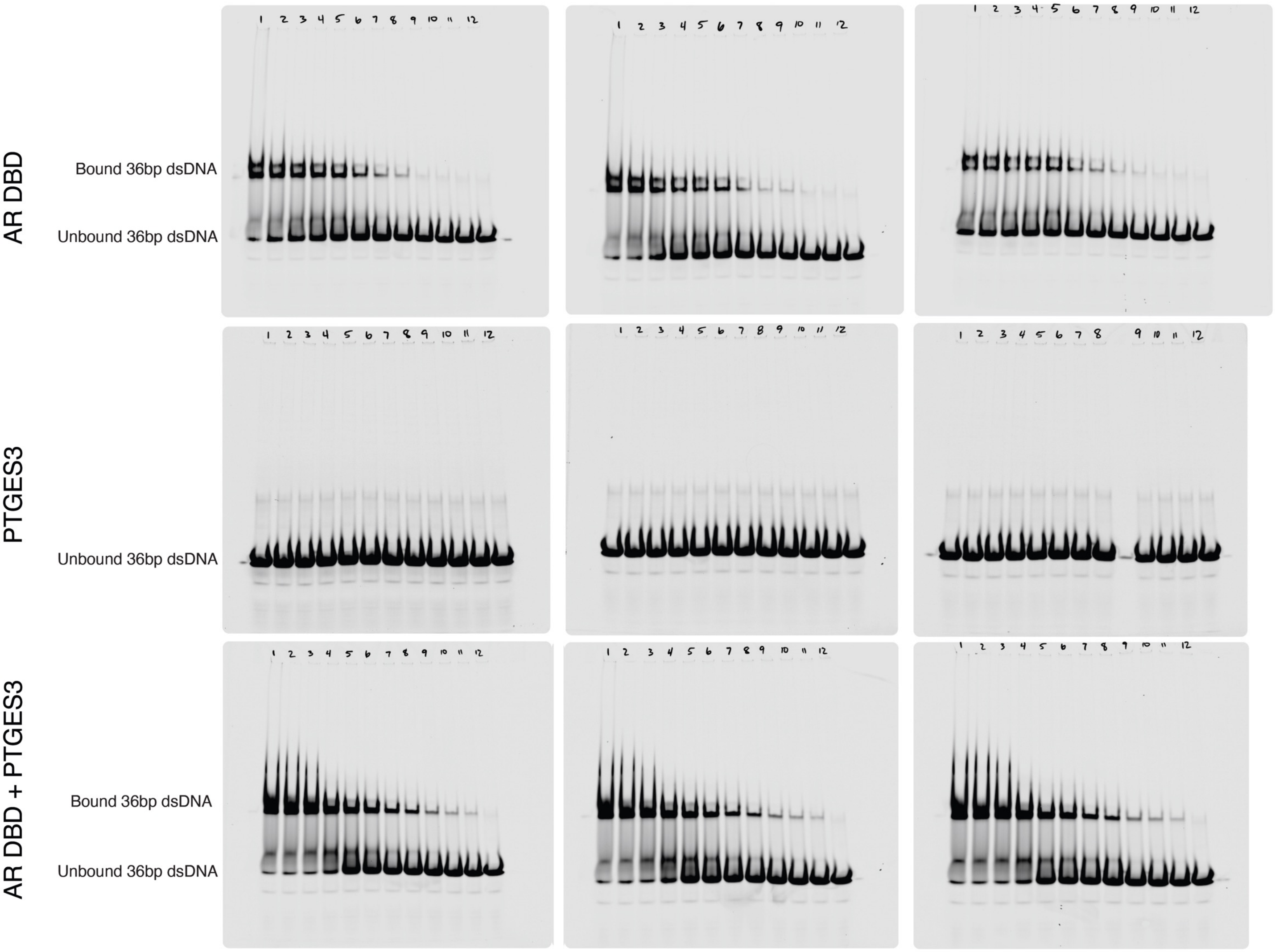

ED Fig.6b

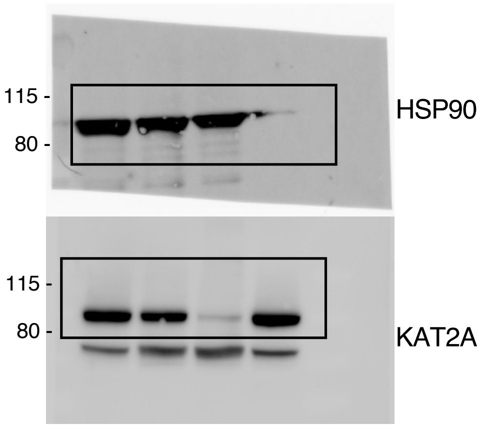

ED Fig.8a

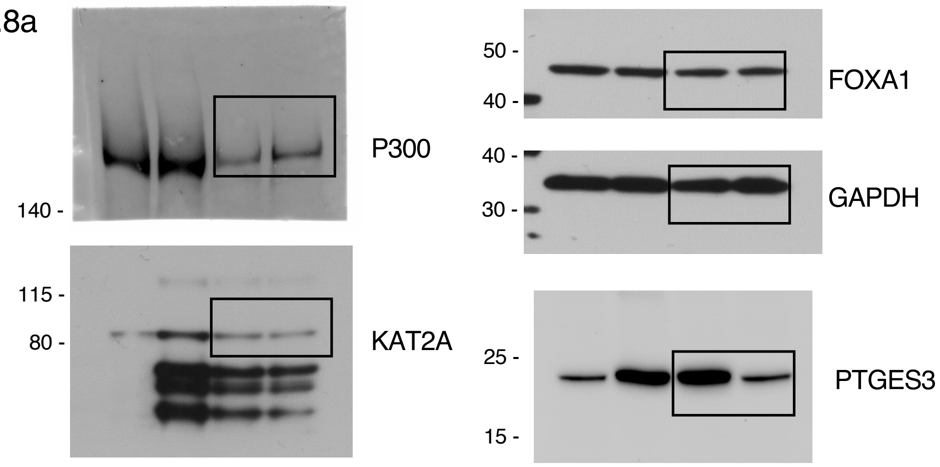

ED Fig.8b

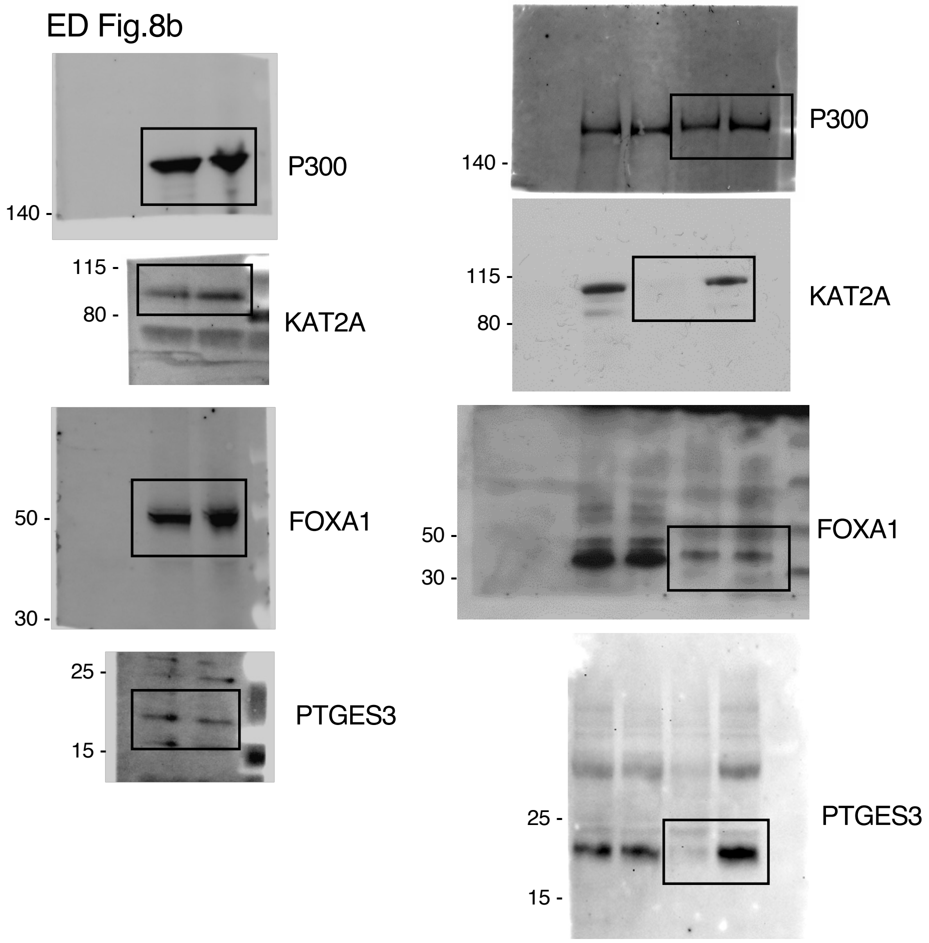

ED Fig.10b

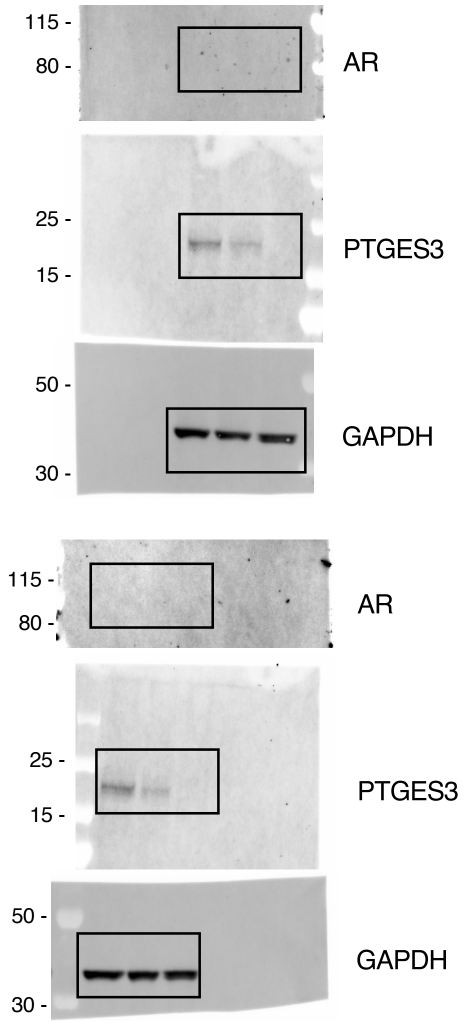

ED Fig.10f

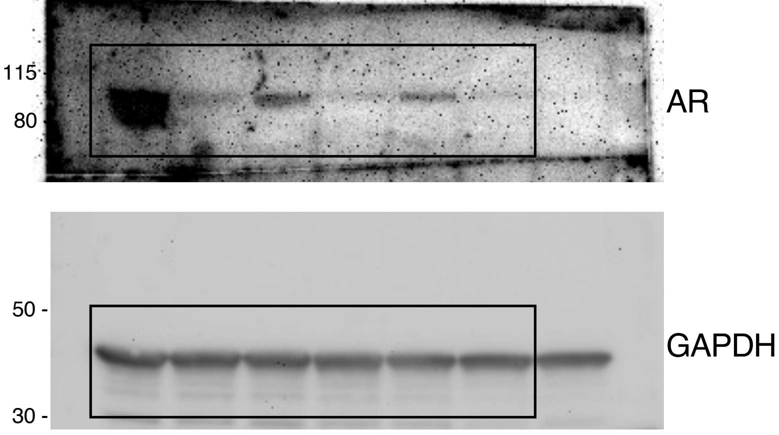

Supplement: Supplementary file 8 — Unprocessed western blots. [file 41588_2025_2388_MOESM8_ESM.pdf]
